# Cognitive Deficits, Changes in Synaptic Function, and Brain Pathology in a Mouse Model of Normal Aging

**DOI:** 10.1523/ENEURO.0047-15.2015

**Published:** 2015-10-15

**Authors:** Martin Weber, Tiffany Wu, Jesse E. Hanson, Nazia M. Alam, Hilda Solanoy, Hai Ngu, Benjamin E. Lauffer, Han H. Lin, Sara L. Dominguez, Jens Reeder, Jennifer Tom, Pascal Steiner, Oded Foreman, Glen T. Prusky, Kimberly Scearce-Levie

**Affiliations:** 1 Department of Neuroscience, Genentech, South San Francisco, California 94080; 2 Department of Psychiatry, University of California, San Diego, La Jolla, California 92093; 3 Burke Medical Research Institute, White Plains, New York, 10605; 4 Department of Pathology, Genentech, South San Francisco, California 94080; 5 Department of Bioinformatics, Genentech, South San Francisco, California 94080; 6 Department of Physiology and Biophysics, Weill Cornell Medical College, Burke Medical Research Institute, White Plains, New York 10605

**Keywords:** age, c-Fos, cognition, gliosis, mice, synaptic function

## Abstract

Age is the main risk factor for sporadic Alzheimer’s disease. Yet, cognitive decline in aged rodents has been less well studied, possibly due to concomitant changes in sensory or locomotor function that can complicate cognitive tests. We tested mice that were 3, 11, and 23 months old in cognitive, sensory, and motor measures, and postmortem measures of gliosis and neural activity (c-Fos). Hippocampal synaptic function was also examined. While age-related impairments were detectable in tests of spatial memory, greater age-dependent effects were observed in tests of associative learning [active avoidance (AA)]. Gross visual function was largely normal, but startle responses to acoustic stimuli decreased with increased age, possibly due to hearing impairments. Therefore, a novel AA variant in which light alone served as the conditioning stimuli was used. Age-related deficits were again observed. Mild changes in vision, as measured by optokinetic responses, were detected in 19- versus 4-month-old mice, but these were not correlated to AA performance. Thus, deficits in hearing or vision are unlikely to account for the observed deficits in cognitive measures. Increased gliosis was observed in the hippocampal formation at older ages. Age-related changes in neural function and plasticity were observed with decreased c-Fos in the dentate gyrus, and decreased synaptic strength and paired-pulse facilitation in CA1 slices. This work, which carefully outlines age-dependent impairments in cognitive and synaptic function, c-Fos activity, and gliosis during normal aging in the mouse, suggests robust translational measures that will facilitate further study of the biology of aging.

## Significance Statement

While age is the main risk factor for many dementias, age-related cognitive decline in rodents remains challenging to study due to concomitant changes in sensory and locomotor function. We have rigorously assessed the potential role of such confounds and identified age-dependent changes in gliosis, synaptic physiology, and behavior that hold promise for replication across laboratories. These findings enable task selection for the evaluation of cognitive enhancers and as probes into the basic biology of age-related cognitive decline.

## Introduction

Age is the most common risk factor for cognitive decline in humans (Prince et al., 2013). This applies to many dementias of old age such as late-onset Alzheimer’s disease (AD), Lewy body, vascular, or frontotemporal dementia; dementias of young age such as early-onset AD and Down’s syndrome (Wu et al., 2012; Vieira et al., 2013); as well as cognitive decline in healthy, nondemented individuals (Caserta et al., 2009). Therefore, models of normal aging can provide critical insight into the processes of cognitive decline. Progress has been made toward establishing such models, initially mostly in rats, and more recently in mice (Erickson and Barnes, 2003; Kennard and Woodruff-Pak, 2011). Many behavioral studies on aging have focused on relatively isolated measures of cognitive functions. Yet more studies are needed that use a range of assessments to enable careful cross-validation of individual measures and comparisons of effect sizes and sensitivities between measures. Further, few studies have simultaneously assessed the potential impact of age-related changes in sensory and motor function on presumed cognitive measures (Caserta et al., 2009) or adjusted behavioral protocols to mitigate the effects of these noncognitive changes (Dolgin, 2013). This may be of particular concern with C57BL/6 mice, one of the most common background strains for genetic models, yet one in which hearing deficits emerge with age (Johnson et al., 1997; Francis et al., 2003). Finally, the value of behavioral phenotypes can be greatly enhanced if they are complemented by robust physiological and postmortem phenotypes that elucidate the underlying biology and support the design of more robust preclinical studies with multiple efficacy endpoints.

Here, we have conducted immunohistochemistry (IHC) for gliosis using GFAP and CD68 as markers of activated astrocytes and microglia, respectively, with the goal of assessing whether putative changes in behavior reliably coincide with changes in the inflammatory environment in aged mice. Since such changes can modulate neuronal and synaptic homeostatic processes, we measured basal and novelty-induced c-Fos induction in the dentate gyrus (DG), and synaptic physiology in the CA1 of the hippocampal formation (HPF). We have carefully characterized three age groups of C57BL/6 mice in behavioral tests. Sensory capabilities and general locomotor function were assessed, and behavioral protocols were adjusted to evaluate whether the observed effects in putatively cognitive tasks were indeed likely to be cognitive in nature, rather than the result of age-related sensory deficits or other nonspecific age-related factors. Aged mice had strong active avoidance (AA) deficits that were independent of changes in hearing or exploratory behavior. Spatial learning deficits were mild and partly linked to altered exploratory activity, while minimal age-related changes were seen during fear conditioning (FC) testing. Aged mice showed increased GFAP and CD68 staining in the HPF, reduced neuronal activation, and alterations in synaptic physiology. These findings provide a strong, well defined phenotype to explore the biology of age-dependent cognitive changes in the future.

## Materials and Methods

### Animals

A total of 110 female C57BL/6 mice were obtained from The Jackson Laboratory and were aged in an Association for Assessment and Accreditation of Laboratory Animal Care International-approved housing facility. Lights-on was at 5:30 a.m. and a 14/10 h light/dark schedule was used. Water and food were available *ad libitum*. Mice were single housed prior to behavioral testing. The bulk of the behavioral studies were conducted in 54 mice that were 3 months (“young,” *n* = 17), 11 months (“middle-aged,” *n* = 17), or 23 months (“aged,” *n* = 17) old at the beginning of testing. Testing was completed within a time period of 75 d, such that mice were ∼5, 13, and 25 months old during the later phases of behavioral testing. Mice were carefully monitored for any signs of sickness throughout the study. For behavioral testing, mice were split into three cohorts (cohorts 1–3). Each of these cohorts consisted of mice from all three age groups to enable direct comparisons of the age groups. A separate cohort of 16 young (almost 4 months old) and 20 aged mice (slightly over 19 months old) was used for tests of visual AA (cohort 4). Three weeks after AA testing, when these mice were a little over 4 months and not quite 20 months old, respectively, optokinetic tests were conducted. For simplicity, they are referred to as 4 and 19 month olds throughout the article. All testing occurred during the light phase. Another cohort of five young (∼4 months old) and five aged (∼21 months old) female mice were used for electrophysiological assays (cohort 5). Animal studies were approved by the Institutional Animal Care and Use Committee and were conducted in accordance with the National Institutes of Health *Guide for the Care and Use of Laboratory Animals*.

### Behavioral tests

#### General

**Figure 1.**
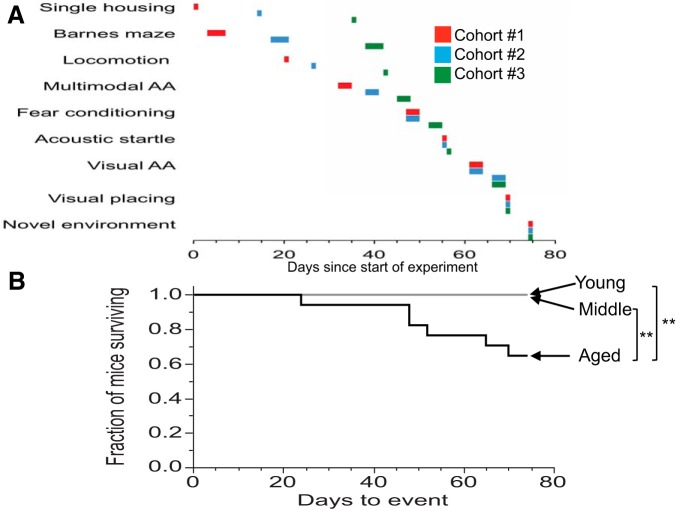
***A***, ***B***, Sequence and timing of *in vivo* experiments (***A***) and time course of mortality in aged mice (***B***). ***A***, Three cohorts of mice, each containing mice from each of the three age groups, were used for the majority of behavioral testing. Testing was completed within 75 d after single housing. Testing began with Barnes maze training, followed by tests of locomotion, multimodal AA (in half of the mice), FC, acoustic startle, visual AA (in the other half of the mice that were previously not tested in multimodal AA), visual placing, and, finally, NE. Mice were sacrificed immediately after the NE experiment. ***B***, The time until a mouse died or had to be removed from the study due to health reasons is shown relative to the beginning of the study when mice were housed singly (see **A**). **p < 0.005.

Throughout all behavioral experiments, conditions were counterbalanced to avoid any systematic effects of recording chambers, arenas, or time of the recordings between the different age groups. For all behavioral tests, except optokinetic testing, mice were acclimated in an outer room adjacent to housing and experimental rooms (AA, FC, acoustic startle) or in the actual experimental room (Barnes maze, locomotor activity, visual placing, and eyeblink tests) for at least 20 min prior to the beginning of the experiment. For optokinetic testing, no explicit acclimation period was used. Experimental chambers and arenas were cleaned using water or ethanol-containing wipes after each animal was tested. After at least 3 d of single housing, mice from cohorts 1, 2, and 3 were submitted to the sequence of behavioral tests shown in Figure 1. Testing began with the Barnes maze and open field assessment of locomotor activity. To avoid any systematic sequence effects between AA and FC tests, the sequence of AA and FC was counterbalanced between mice: approximately half of the mice first underwent (multimodal) AA before being tested in the FC assay, while the remaining half of the mice were tested in the FC assay without prior AA testing. FC testing was followed by acoustic startle testing for all mice. Those mice that were not previously exposed to AA testing subsequently underwent visual AA testing. Visual placing and eyeblink responses were assessed next. Finally, a subset of mice was exposed to a novel environment (NE) immediately before tissue harvesting. For cohort 4, all mice underwent visual AA testing, followed by optokinetic testing 3 weeks later.

#### Locomotor activity

The experimental room was illuminated, providing **∼**300 lux in the middle of the locomotor chambers. PAS open-field recording systems (San Diego Instruments) consisted of transparent thermoplastic cages (width, 40.5 cm; length, 40.5 cm; height, 38 cm) surrounded by a locomotor frame and a rearing frame fitted with infrared (IR) beams to record horizontal locomotor activity (3 cm above the floor) and rearing activity (7.5 cm above the floor), respectively. The total number of beam breaks for both locomotor activity and rearing was calculated for a total of 15 min.

#### Visual placing and eyeblink responses

Visual placing tests were conducted as outlined in the article by Crawley (2007). Briefly, mice were held by the tail and lowered toward the table surface. The presence or absence of an anticipatory forepaw extension in proximity to the table before contact of the whiskers or any other body part was used as the readout. Tests were repeated up to three times per mouse. An absence of a forepaw extension was scored if no response was observed in any of these individual trials. Eyeblink responses were used as additional criteria if forepaw extension was not detected in an animal. An absent eyeblink response was scored if the mouse failed to blink in response to a cotton swab approaching each of the eyes without touching the whiskers or eyelids. A single trial was used for each of the eyes.

#### Barnes maze

A Barnes maze platform for mice (Stoelting) was used. Light intensity was **∼**1000 lux in the middle of the platform. The platform was surrounded by diverse extramaze cues on the walls. The maze consisted of a white, horizontal circular platform (91 cm in diameter, elevated 90 cm above the floor) that contained 20 holes (each of which was 5 cm in diameter). Each hole was located 5 cm from the edge of the maze and 13 cm from each adjacent hole (measured from the center of the holes). Underneath one target hole, a dark escape tunnel (25 × 6 × 5 cm) was located. TopScan (Cleversys) software recorded the distance travelled (in centimeters) and velocity of movement (in centimeters per second), as well as the number of visits for each individual hole. Settings were optimized to detect the nose of the mouse as the center for tracking. Maze training and testing were completed on 4 consecutive days, beginning with 1 d of pretraining, followed by 2 d of regular Barnes maze training, followed by a single day on which first probe testing and later reversal training were conducted. During pretraining trials, access to the maze platform was restricted to a small corridor of the platform that included only the escape hole. A polyvinyl chloride (PVC) ring (10 cm in diameter by 8 cm in height) served as the starting location for the mice. During each of the pretraining, training, or probe trials, individual mice were placed into the PVC ring in the center of the platform for a brief period of time, and the trial began by removing the ring to allow the mouse to freely explore the maze. If the mouse did not access the escape tunnel within 60 s, it was gently pushed toward the escape hole. After the mouse had entered the escape tunnel, it was allowed to stay there for several seconds. Pretraining was completed if a mouse entered the escape tunnel twice in a row by itself, or after a maximum of four trials, whichever came first.

During the 2 training days, mice underwent four training trials per day in which the entire maze platform was accessible and the location of the escape hole was kept constant. Each trial was stopped automatically after a maximum of 180 s, after the mouse had entered the escape hole or hovered for ∼3 s above the escape hole, whichever came first. In the 180 s probe test, the escape tunnel was removed from underneath the escape hole and the pattern of exploration of the animal was recorded. After completion of the probe trial, the escape tunnel was again attached to the platform, but at a position opposite to the previous location of the target hole so that the ability to learn an entirely new location could be tested (reversal). Reversal training consisted of three trials. All other aspects of reversal training were similar to that of the regular training phase.

#### Fear conditioning

FC chambers for mice (30 × 24 × 24 cm measured inside of the chamber) located inside of sound-attenuating cubicles (Med Associates) were used. Each chamber was equipped with a house light, an IR light, a speaker that was used to deliver white noise during the training and cued phases of the task, and a near-IR camera to record the movement and/or freezing of the mice. The floors of the compartments were equipped with stainless steel metal grids that were connected to a shock generator. Hardware and data acquisition were controlled by Video-Freeze software (Med Associates). Video data were acquired at a rate of 30 frames/s; the observation interval was 15 frames or 0.5 s; and the observation duration was 3 frames or 0.1 s. The motion threshold was set to 20 (artificial units). Data were acquired with the discrete method: if the motion index was <20 (artificial units) throughout a recording period, freezing was registered. The percentage of freezing was defined as the percentage of recording periods in which freezing occurred relative to the total number of observation periods. The experimental task consisted of 3 consecutive days, beginning with training on day 1 and a contextual test on day 2, followed by a cued test on day 3. On days 1 and 2, the house lights were on and the metal grids served as the floor. On day 3, the context of the chamber was altered by switching off the house lights, covering the grid floor with a plastic inlay, altering the shape of the chamber walls by another insert, and changing the scent of the chamber with a small amount of 1% acetic acid (Mallinckrodt Chemicals). The recording settings were readjusted to account for the different lighting and background conditions for the camera on day 3. The system was calibrated using camera recordings from empty chambers before the mice were placed inside. FC training began with 3 min of baseline recording, followed by two trials, each consisting of a 2 s shock (0.7 mA), which served as an unconditioned stimulus (UCS) and 30 s of white noise [∼90 A-weighted dB (dBA); background noise, ∼65 dBA measured ∼3 cm above the grid floor], which initially served as the neutral stimulus (NS) or conditioned stimulus (CS). NS or CS coterminated with the UCS. The two trials were separated by an intertrial interval (ITI) of 90 s (offset to onset), and the second trial was followed by another 90 s of recording to measure freezing at the end of the training period (see Fig. 4A). During contextual FC, each mouse was reintroduced into the same unaltered chamber in which FC training took place for a recording period of 5 min during which neither white noise nor shock was given. Cued FC testing was conducted in different chambers for each individual mouse and in an altered context (see above). Testing consisted of 3 min of baseline recording, followed by 3 min of white noise without shock. The percentage of time spent freezing was calculated for each of these experimental phases. To evaluate whether pain responsiveness differed between groups, the movement intensity before, during, and after the shocks of the training phase was recorded using motion index data (artificial units) with a 2 s resolution and was averaged across the two trials (see Fig. 4B). For data analysis, the average motion index across the five 2 s bins prior to the shocks served as (pre-UCS) baseline relative to the response during the actual 2 s UCS (during UCS).

#### Acoustic startle

Startle chambers (SR-Lab, San Diego Instruments) consisted of a thermoplastic cylinder 3.8 cm in inner diameter resting on a 12.5 × 20.5 cm thermoplastic frame within a ventilated enclosure. Noise bursts and background noise were presented via a speaker mounted ∼25 cm above the cylinder. A piezoelectric accelerometer mounted below the frame detected and transduced motion from within the cylinder. Stimulus delivery was controlled by the SR-LAB microcomputer and interface assembly, which also rectified and recorded stabilimeter readings. Sixty-five 1 ms readings were collected beginning at stimulus onset. Startle amplitude was defined as the average of the 65 readings. Mice were placed in the startle chambers for a 5 min acclimation period with 65 dBA background noise and then exposed to a series of trial types. All active trials consisted of bursts of white noise of 40 ms duration of varying SPL intensities above background. Interspersed between all active trials were trials in which no stimulus was presented, but cage displacement was measured (NOSTIM trials). The session began and ended with a block of three trials of 55 dB SPL intensity above background. Between these trials was one block of trials with eight of each of the following trial types that were presented in pseudorandom order: 4, 8, 12, 16, 20, 24, 28, 35, 42, 49, or 55 dB above background. The ITI ranged from 8 to 22 s and averaged 15 s. NOSTIM trials were not included in the calculation of ITI. Total session duration was 22.5 min. To extract the most meaningful data out of any intensity-dependent effects while avoiding multiple-comparison problems with this factor and its nine levels, slopes of the movement intensity over stimulus intensity were calculated for each individual mouse using linear regression analysis.

#### Active avoidance

Two-way avoidance chambers for mice located inside of sound-attenuating cubicles (Med Associates) were used. Each chamber contained two compartments (each one 21 × 16 × 25 cm, measured inside of the chamber) that were connected by an auto-guillotine door. Each compartment was equipped with one stimulus light and one stimulus tone generator, as well as four IR light beams located 2.5 cm above the grid floor that were aligned in parallel across the long axis of the chamber to locate the mouse. The floors of the compartments were equipped with stainless steel metal grids that were connected to a shock generator. Hardware and data acquisition were controlled by Med-PC software (Med Associates). The experimental task was modified based on a protocol by Foldi et al. (2011). Two different AA tasks, a multimodal and a visual version, were designed to vary the salience of the acoustic stimuli. For each of the two tasks, AA training was conducted on 2 consecutive days such that each animal received two training sessions, each consisting of 100 trials. Each session began by placing a mouse into one of the two compartments. The starting side was counterbalanced across mice. The door between the compartments remained closed, and all lights inside of the enclosure were off for the duration of a 5 min acclimation period. For multimodal AA testing, each trial began with the opening of the guillotine door, and a stimulus light (∼10 lux at floor level) and tone stimulus (∼73 dBA) came on. Background noise was ∼68dBA at floor level. If the mouse crossed into the second compartment at any time within 5 s of stimulus presentation, the door was closed; the light and the tone were switched off immediately; an avoidance response was recorded; and the ITI began. If, however, the mouse did not cross within 5 s, a shock (0.3 mA, 2 s) was delivered through the grid floor. If the mouse crossed within 2 s, the shock was stopped; the door was closed; the light and the tone were switched off; an escape response was recorded, and the ITI began. If, however, the mouse did not cross within 2 s, the shock stopped; the door was closed; the light and the tone were switched off; an escape failure was recorded, and the ITI began. The ITIs ranged from 25 to 55 s and averaged 40 s. After the ITI was completed, the next trial was started. This trial–ITI sequence was continued until 100 trials were completed. The visual AA task was identical to the multimodal version with the exception that only the stimulus light, but not the stimulus tone, was presented during each of the trials. Despite the absence of an explicit tone, a residual acoustic signal could be tied to the opening and closing of the doors in the experimental chambers. The percentage of avoidance, percentage of escapes, and percentage of escape failures were calculated using average values obtained from only five blocks of 20 consecutive trials, thereby enabling ANOVA at a limited number of factor levels for 100 individual trials. To obtain an index that better reflects the learning progress with increasing number of trials, the percentage of avoidance was separately calculated using blocks of five trials. These data were then used as the basis for linear regression analysis to obtain slopes (percentage of avoidance/blocks of five trials) for each individual mouse and training day. Since the percentage of avoidance, percentage of escapes, and percentage of escape failures represent all the possible response types, their total sum equals 100. Cohort 4 only underwent the visual version of the AA task. Data from one (aged) mouse in cohort 4 had to be excluded from AA analysis due to equipment failure.

#### Novel environment

All mice were left undisturbed in their home cages for 3 d prior to the experiment. Half of the mice were then randomly assigned to the home cage condition in which they continued to stay undisturbed in their home cages until tissue harvesting. The other mice were directly transferred from their home cages to the NE condition in which they remained for the 2 h preceding tissue harvest. The NE consisted of a large, transparent macralon cage (43 × 22 × 20 cm) that contained a diverse set of items and stimuli that were new to the mice, including ALPHA-dri bedding material (Shepherd Speciality Papers), a small pool of water, fruit loops, a banana slice, a scented cotton swab, a paper cage card, and other plastic and paper objects.

### Behavioral measures of visual function

Spatial thresholds for optokinetic tracking were measured using a virtual optokinetic system (Prusky et al., 2004). One spatial frequency (SF) threshold, and contrast thresholds at six SFs [0.031, 0.064, 0.092, 0.103, 0.192, and 0.272 cycles/degree (c/d)] were measured through each eye (Prusky et al., 2004; Prusky et al., 2006) in each of two testing sessions conducted over 2 d. A vertical sine wave grating was projected as a virtual cylinder in 3-D coordinate space on computer monitors (mean white = 247.604 cd/m^2^; mean black = 0.260 cd/m^2^) surrounding a testing arena. Mice were placed one at a time on an elevated platform at the epicenter of the arena and allowed to move freely. An experienced observer used a live overhead video image of the arena to view the animal and track a position between the eyes in real time, using a computer mouse and a crosshair superimposed on the frame. The X–Y positional coordinates of the crosshair centered the hub of the virtual cylinder enabling. When the cylinder was rotated and the animal engaged in reflexive head and neck movements to track the rotation, it was judged that the animal could distinguish the grating. Homogeneous gray was presented on the cylinder at the beginning of each testing session. For threshold measures of SF, the experimenter waited until the mouse was stationary, at which time gray was replaced with a low-SF, high-contrast sine wave grating of the same mean luminance, drifting in one direction. The mouse was assessed for tracking behavior for a few seconds, after which the stimulus returned to gray. The procedure was repeated until unambiguous examples of tracking were observed. The SF of the grating was then increased incrementally, with multiple tests at each SF, until the highest SF for tracking was identified. Thresholds through each eye were measured independently by altering the direction of cylinder rotation, since only temporal-to-nasal motion in the visual field evokes tracking (Douglas et al., 2005). Contrast thresholds were generated using similar procedures, except that grating contrast was systematically varied, and a contrast sensitivity function was produced by calculating at each SF a Michelson contrast from the screen luminance (maximum − minimum)/(maximum + minimum), and plotting the reciprocal of the threshold. All experiments were conducted using a cylinder rotation rate of 12°/s. Reported measures are average values of the two eyes over the two testing sessions. The experimenter had no prior knowledge of the age or previous treatment of the animals.

### Tissue harvesting

Mice from the different experimental conditions and age groups were sacrificed in a pseudorandom order. Tissue harvesting was conducted immediately after the completion of the exposure to the novel environment or home cage condition. Briefly, small groups of three to four mice were deeply anesthetized using 2.5% 2,2,2-tribromoethanol (0.5 ml/mouse; Sigma-Aldrich), immediately transferred to a necropsy room, and transcardially perfused with PBS. The brain was extracted and the (left/right) hemibrain was post-fixed in 4% paraformaldehyde (Electron Microscopy Sciences) in PBS for 48 h at 4°C on a shaker and subsequently transferred into a 30% sucrose (Sigma-Aldrich) solution in PBS and stored at 4°C until tissue sectioning.

### Brain sectioning and immunohistochemistry

Fixed hemibrains were sectioned sagittally in 35 μm sections on a freezing microtome (SM2000R, Leica Microsystems). Free-floating sections were blocked in goat serum and incubated in primary antibody overnight at 4°C, followed by incubation in secondary antibody for 1 h. For GFAP staining, 1:500 mouse anti-GFAP (Thermo Scientific) was the primary antibody, and 1:500 goat anti-mouse (Vector Laboratories) the secondary antibody. For CD68 staining, 1:800 rat anti-CD68 (AbD Serotec) was the primary antibody, and 1:500 rabbit anti-rat (Vector Laboratories) was the secondary antibody. For c-Fos staining, 1:10,000 rabbit anti-c-Fos (Ab-5, Calbiochem) was the primary antibody, and 1:200 goat anti-rabbit (Vector Laboratories) was the biotinylated secondary antibody. After exposure to the secondary antibody, sections were incubated in avidin–biotin complex (ABC Reagent, Vector Laboratories) reagent and developed in 3,3' diaminobenzidine hydrochloride (Sigma-Aldrich) solution. Stained sections were mounted onto slides with 1% gelatin (Bio-Rad Laboratories), dried overnight, and then dipped in xylene (EMD Chemicals) for 5 min before coverslipping with Cytoseal 60 (Thermo Scientific).

### Imaging and image analysis

Whole slide images of immunolabeled brain sections were acquired using the Nanozoomer (Olympus) system at 200× magnification. Up to four parasagittal sections that matched lateral levels of 2.525, 2.35, 1.75, and 1.35 mm (relative to the midline of the brain) from the *Allen Mouse Brain Reference Atlas* [version 1, 2008, sagittal (http://mouse.brain-map.org/static/atlas] were selected for analysis. Regions of interest (ROIs) were manually outlined for the DG, isocortex, and HPF (including the DG), as defined by the *Allen Mouse Brain Reference Atlas*. Positive pixel areas for GFAP and CD68 in the HPF were automatically analyzed in MATLAB version 7.14 (MathWorks) using an intensity threshold set to detect dark stained areas only. The threshold was applied consistently across all images, and all segmentation overlays were visually inspected to ensure adequate detection. An additional minimum size cutoff of 135 and 13 μm2 was applied to detect clustered and enlarged cells for the GFAP and CD68 staining, respectively. Clustered (GFAP) and enlarged (CD68) staining areas were normalized to the total ROI area in order to obtain the percentage of the stained area. The average size (in mm^2^), density (# of objects/mm^2^), and shape factor of the stained objects were determined for each ROI. The shape factor was calculated from the Equation 4, *pi*area/perimeter^2, where a perfect circle would have a value of 1 and an elongated thin line would have a value approaching 0. Averages were calculated from the three to four sections for each animal. Image analysis, quality control, and selection of sections and ROIs were performed blind to the experimental conditions and age of the animals. GFAP data from two mice (one young, one middle-aged mouse) were excluded due to evidence of vessel staining, which indicated poorly perfused tissue. One additional aged mouse had to be excluded from GFAP analysis, and two excluded from CD68 analysis due to poor tissue quality. In addition, since one middle-aged mouse did not have any detectable GFAP-positive clusters, this mouse had to be excluded from the analyses of the average GFAP-positive cluster size and shape factor, but it is included in the calculation of GFAP-positive cluster density. Cells positive for c-Fos staining in the DG were counted manually. An observer blinded to the experimental group and condition performed all manual cell counting, section selection, and ROI definition. The isocortex exhibited more c-Fos-positive cells and was automatically analyzed in MATLAB. Briefly, the number of c-Fos-positive nuclei was counted using color thresholds and minima-controlled watershed segmentation followed by morphological operations. A minimal size exclusion of 7 μm^2^ was applied to remove small bits of noncellular stained area. Sections from one aged mouse were excluded from c-Fos analysis due to tissue tearing and poor section quality. A board-certified pathologist reviewed the images and data independently.

### Electrophysiology

The 400-µm-thick hippocampal slices were prepared with a vibrating sectioning system (Leica) and were recorded in oxygenated artificial cerebrospinal fluid (ACSF) containing the following (in mm): 127 NaCl, 2.5 KCl, 1.3 MgSO4, 2.5 CaCl2, 1.25 Na2HPO4, 25 NaHCO3, and 25 glucose. Slices were prepared in ice-cold oxygenated ACSF with the MgSO4 concentration elevated to 7 mm, and NaCl was replaced with 110 mm choline and 11.6 mm Na-ascorbate, and 3.1 mm Na-pyruvate was added. During recovery from slicing, slices were incubated at 34°C for 45 min before being stored at room temperature. Field recordings of EPSPs were measured from the stratum radiatum of CA1 in response to stimulation of Schaffer collateral inputs. Input–output (I–O) relationships, paired-pulse ratio (PPR), and LTP measurements were performed as described previously (Hanson et al., 2014). Briefly, for I–O measures, stimulation intensity was logarithmically increased with each trial (up to 1 mA). For PPR measures, the duration of the interstimulus interval (ISI) was doubled with each trial; beginning with 25 ms up to 1600 ms. An intertrial interval of 20 s was used. LTP was induced using theta burst stimulation (TBS) consisting of five pulses at 100 Hz repeated 10 times at 200 ms intervals. LTP was first induced using a single bout of TBS, followed 20 min later by two bouts of TBS (separated by 20 s), and another 20 min later by three bouts of TBS (separated by 20 s).

### Data analysis

Data were analyzed using mixed-design or between-subjects ANOVAs, as appropriate. *Post hoc* comparisons were made using Tukey’s HSD test in order to account for more than two comparisons between factors or factor levels, or Student’s *t* test for two comparisons only. The biological units of observations for analyses were animals for behavioral and IHC measures, and brain slices for electrophysiological measures. Morbidity data were assessed via Kaplan–Meier survival estimates followed by generalized Wilcoxon χ^2^ tests for the estimated survival functions between age groups. The collection of tissue from the mice at the end of the study served as censor criteria. Correlational analyses were based on Pearson’s product-moment correlation calculations. Box plots were used in which the midline corresponds to the median value, and the upper and lower margins of the boxes correspond to the 25% and 75% quartiles. The lengths of the whiskers on the graph mark the outmost data points within a 1.5-fold interquartile distance beyond the 25% or 75% quartile. Values beyond these calculated limits are defined as outliers (shown as circles). Line graphs with mean ± SEM values are shown to best depict data with five or more levels in one factor, such as time courses. Where feasible, significant *p* values for the critical age effects are shown in the figures. Observed *p* values are listed in Table 1 rather than observed power values due to the complications of power analyses with most statistical software packages when it comes to *post hoc* tests such as Tukey’s test that address the multiple comparisons problem. Since observed power is the result of a 1:1 mathematical function of the *p* value, the observed *p* values that were reported here give identical information as observed power. A (two-tailed) α of 0.05 was used. Analyses and graphs were generated using JMP version 10.02 (SAS Institute).

**Table 1.** Statistics

		Data structure	Test	Exact *p* value (or estimated *n*)	*N*
a	Animal morbidity	Kaplan–Meier curves, one-factor, btw (age)	χ^2^	0.001 (age)	17 y; 17 m; 17 a
b	Horizontal BB	One-factor, btw (age)	ANOVA	0.27 (age)	17 y; 17 m; 17 a
c	Rearing	One-factor, btw (age)	ANOVA	0.24 (age)	17 y; 17 m; 17 a
d	% BB in center	One-factor, btw (age)	ANOVATukey’s	0.004 (age) 0.004 (y vs a), 0.027 (y vs m), 0.73 (m vs a)	17 y; 17 m; 17 a
e	Barnes maze training (distance)	Two-factor, mixed-design: btw (age) and win (trial)	ANOVATukey’s	0.018 (age), <0.0001 (trial), 0.67 (age × trial) 0.015 (y vs a), 0.15 (y vs m), 0.55 (m vs a)	17 y; 17 m; 16 a
f	Barnes maze training (velocity)	Two-factor, mixed-design: btw (age) and win (trial)	ANOVATukey’s	0.044 (age), <0.0001 (trial), 0.006 (age × trial) 0.25 (y vs a), 0.037 (y vs m), 0.64 (m vs a)	17 y; 17 m; 16 a
g	Barnes maze probe (bouts; all age groups)	Two-factor, mixed-design: btw (age) and win (hole type)	ANOVATukey’s	0.035 (age), <0.0001 (hole type), 0.053 (age × hole type) Age: 0.047 (y vs a), 0.087 (y vs m), 0.95 (m vs a); Hole type: <0.0001 Target hole: 0.043 (y vs a), 0.13 (y vs m), 0.84 (m vs a) Nontarget hole: 0.77 (y vs a), 0.15 (y vs m), 0.47 (m vs a)	17 y; 17 m; 16 a
h	Barnes maze probe (Δ bouts and % BB)	Two variables:Δ bouts (at target – average nontarget hole) and % BB in center in aged mice	Pearson’s *r*	*p* = 0.046	16 a
i	Barnes maze reversal (distance)	Two-factor, mixed-design: btw (age) and win (trial)	ANOVA	0.77 (age), <0.0001 (trial), 0.14 (age x trial)	17 y; 17 m; 16 a
j	Barnes maze reversal (velocity)	Two-factor, mixed-design: btw (age) and win (trial)	ANOVA	0.28 (age), 0.0005 (trial), 0.36 (age × trial)	17 y; 17 m; 16 a
k	FC training (% freezing)	Two-factor, mixed-design: btw (age) and win (training phase)	ANOVA	0.42 (age), <0.0001 (training phase); 0.82 (age × training phase)	17 y; 17 m; 14 a
l	FC training (motion index)	Two-factor, mixed-design: btw (age) and win (time)	ANOVA	0.65 (age), <0.0001 (time),0.67 (age × time)	17 y; 17 m; 14 a
m	FC context test (% freezing)	One-factor, btw (age)	ANOVATukey’s	0.046 (age) 0.89 (y vs a), 0.12 (y vs m), 0.057 (m vs a)	17 y; 17 m; 14 a
n	FC cue test (% freezing)	Two-factor, mixed-design: btw (age) and win (test phase)	ANOVATukey’s	0.31 (age), <0.0001 (test phase), 0.030 (age × test phase)Pre CS: 0.72 (y vs a), 0.73 (y vs m), 0.99 (m vs a) During CS: 0.12 (y vs a), 0.24 (y vs m), m vs a (0.88)	17 y; 17 m; 14 a
o	Acoustic startle(movement intensity)	Two-factor, mixed-design: btw (age) and win (stimulus intensity)	ANOVATukey’s	<0.0001 (age) <0.0001 (stimulus intensity) <0.0001 (age × stimulus intens.)Age: <0.0001 (y vs a), 0.64 (y vs m), <0.0001 (m vs a)	17 y; 17 m; 14 a
p	Acoustic startle [slope (movement intensity/dB)]	One-factor, btw (age)	ANOVATukey’s	*p* < 0.0001 (age) Age: <0.0001 (y vs a), 0.12 (y vs m), <0.0001 (m vs a)	17 y; 17 m; 14 a
q	Multimodal AA (D1: % AA)	Two-factor, mixed-design: btw (age) and win (blocks)	ANOVATukey’s	<0.0001 (age) <0.0001 (block)<0.0001 (age × block)Age: <0.0001 (y vs a), 0.36 (y vs m), 0.001 (m vs a)	8 y; 8 m; 8 a
r	Multimodal AA (D2: % AA)	Two-factor, mixed-design: btw (age) and win (blocks)	ANOVATukey’s	<0.0001 (age) <0.0001 (block)0.005 (age × block) Age: <0.0001 (y vs a), 0.79 (y vs m), <0.0001 (m vs a)	8 y; 8 m; 8 a
s	Multimodal AA (D1: slope (% AA/block)	One-factor, btw (age)	ANOVATukey’s	Age: 0.002 0.007 (y vs a), 0.98 (y vs m), 0.005 (m vs a)	8 y; 8 m; 8 a
t	Multimodal AA (D2: slope (% AA/block)	One-factor, btw (age)	ANOVATukey’s	Age: 0.024 0.023 (y vs a), 0.72 (y vs m), <0.11 (m vs a)	8 y; 8 m; 8 a
u	Visual AA (D1: % AA)	Two-factor, mixed-design: btw (age) and win (blocks)	ANOVATukey’s	0.036 (age) <0.0001 (block) <0.0001 (age × block)Age: 0.029 (y vs a), 0.30 (y vs m), 0.35 (m vs a)	9 y; 9 m; 6 a
v	Visual AA (D2: % AA)	Two-factor, mixed-design: btw (age) & win (blocks)	ANOVATukey’s	0.002 (age) <0.0001 (block)0.74 (age × block) Age: 0.005 (y vs a), 0.98 (y vs m), 0.004 (m vs a)	9 y; 9 m; 6 a
w	Visual AA (D1: slope (% AA/block)	One-factor, btw (age)	ANOVATukey’s	Age: 0.001 0.003 (y vs a), 0.91 (y vs m), 0.001 (m vs a)	9 y; 9 m; 6 a
x	Visual AA (D2: slope (% AA/block)	One-factor, btw (age)	ANOVATukey’s	Age: 0.80	9 y; 9 m; 6 a
y	Multimodal and visual AA (D1 slope) and % BB in center	1 AA and 1 locomotor variable: D1 slope (% AA/block) and % BB in center	Pearson’s *r*	*p* = 0.54	14 a
z	Visual AA (D1: % AA)	Two-factor, mixed-design: btw (age) and win (blocks)	ANOVA	0.92 (age) <0.0001 (block)0.65 (age × block)	16 y; 19 a
aa	Visual AA (D2: % AA)	Two-factor, mixed-design: btw (age) and win (blocks)	ANOVA	0.099 (age) <0.0001 (block)0.27 (age × block)	16 y; 19 a
ab	Visual AA (D1: slope (% AA/block)	One-factor, btw (age)	ANOVA	Age: 0.39	16 y; 19 a
ac	Visual AA (D2: slope (% AA/block)	One-factor, btw (age)	ANOVA	Age: 0.15	16 y; 19 a
ad	Spatial frequency threshold	One-factor, btw (age)	ANOVA	Age: *p* < 0.0001	16 y; 20 a
ae	Contrast sensitivity	Two-factor, mixed-design: btw (age) and win (spatial frequency)	ANOVA	*p* < 0.0001 (age) *p* < 0.0001 (spatial frequency) *p* < 0.0001 (age × spatial frequency)	16 y; 20 a
af	Visual AA (D1 slope) and spatial frequency threshold	1 AA and 1 spatial frequency variable: D1 slope (% AA/block) and spatial frequency threshold	Pearson’s *r*	*p* = 0.36	19 a
ag	Visual AA (D2 slope) and spatial frequency threshold	1 AA and 1 spatial frequency variable: D2 slope (% AA/block) and spatial frequency threshold	Pearson’s *r*	*p* = 0.85	19 a
ah	Visual AA (D1 slope) and contrast sensitivity at 0.064 c/d	1 AA and 1 contrast sensitivity variable: D1 slope (% AA/block) and contrast sensitivity at 0.064 c/d	Pearson’s *r*	*p* = 0.77	19 a
ai	Visual AA (D2 slope) and contrast sensitivity at 0.064 c/d	1 AA and 1 contrast sensitivity variable: D2 slope (% AA/block) and contrast sensitivity at 0.064 c/d	Pearson’s *r*	*p* = 0.54	19 a
aj	% of clustered GFAP-positive tissue area	Two-factor, btw (age and environment)	ANOVATukey’s	0.005 (age) 0.27 (environment)0.71 (age × environment) Age: 0.049 (y vs a), 0.45 (y vs m), 0.003 (m vs a)	16 y; 16 m; 10 a
ak	Density of GFAP-positive clusters	Two-factor, btw (age and environment)	ANOVATukey’s	0.007 (age) 0.065 (environment)0.67 (age × environment)Age: 0.065 (y vs a), 0.45 (y vs m), 0.005 (m vs a)	16 y; 16 m; 10 a
al	Size of GFAP-positive clusters	Two-factor, btw (age and environment)	ANOVA	0.27 (age) 0.11 (environment) 0.21 (age × environment)	16 y; 15 m; 10 a
am	Shape factor of GFAP-positive clusters	Two-factor, btw (age and environment)	ANOVA	0.35 (age) 0.47 (environment) 0.39 (age × environment)	16 y; 15 m; 10 a
an	% of dark CD68-positive tissue area	Two-factor, btw (age and environment)	ANOVA Tukey’s	<0.0001 (age) 0.132 (environment) 0.088 (age × environment) Age: <0.0001 (y vs a), 0.461 (y vs m), <0.0001 (m vs a)	16 y; 16 m; 9 a
ao	Density of CD68-positive dark objects	Two-factor, btw (age and environment)	ANOVA Tukey’s	<0.0001 (age) 0.17 (environment) 0.18 (age × environment) Age: <0.0001 (y vs a), 0.13 (y vs m), <0.0001 (m vs a)	16 y; 16 m; 9 a
ap	Size of CD68-positive dark objects	Two-factor, btw (age and environment)	ANOVA Tukey’s	<0.0001 (age) 0.12 (environment) 0.75 (age × environment) Age: <0.0001 (y vs a), 0.79 (y vs m), <0.0001 (m vs a)	16 y; 16 m; 9 a
aq	Shape factor of CD68-positive dark objects	Two-factor, btw (age and environment)	ANOVA Tukey’s	<0.0001 (age) 0.60 (environment) 0.24 (age × environment) Age: <0.0001 (y vs a), <0.0001 (y vs m), 0.47 (m vs a)	16 y; 16 m; 9 a
ar	Visual AA (D1 slope) and % GFAP-positive cluster area	1 AA and GFAP measure: D1 slope (% AA/block) and % GFAP-positive cluster area	Pearson’s *r*	*p* = 0.87	10 a
as	Visual AA (D1 slope) and % CD68-positive dark area	1 AA and CD68 measure: D1 slope (% AA/block) and % CD68-positive dark area	Pearson’s *r*	*p* = 0.90	10 a
at	Number of c-Fos-positive cells (DG)	Two-factor, btw (age and environment)	ANOVA Tukey’s	0.001 (age) 0.001 (environment) 0.30 (age × environment) Age: 0.004 (y vs a), 0.002 (y vs m), 0.97 (m vs a) Environment: 0.001	17 y; 17 m; 10 a
au	Number of c-Fos-positive cells (isocortex)	Two-factor, btw (age and environment)	ANOVA Tukey’s	0.98 (age) <0.0001 (environment) 0.56 (age × environment) Environment: <0.0001	17 y; 17 m; 10 a
av	Number of c-Fos-positive cells (DG) and % BB in center	1 c-Fos and 1 locomotor variable: number of c-Fos-positive cells (DG) and % BB in center	Pearson’s *r*	*p* = 0.47	10 a
aw	I–O relationship (EPSP slope)	Two-factor, mixed-design: btw (age) and win (stimulus intensity)	ANOVA	0.021 (age), <0.0001 (stimulus intensity), <0.0001 (age × stimulus intensity)	20 y, 5 y; 30 a, 5 a
ax	PPR (p2/p1)	Two-factor, mixed-design: btw (age) and win (ISI)	ANOVA	0.024 (age), <0.0001 (ISI), <0.015 (age × ISI)	22 a, 5 a; 30 a, 5 a
ay	LTP (EPSP slope)	Two-factor, mixed-design: btw (age) and win (number of TBSs)	ANOVA	0.11 (age), <0.0001 (number of TBSs), <0.89 (age × number of TBS)	12 y, 5y; 17 a, 5 a
az	LTP (EPSP slope; responses to 1 TBS pulse only)	One-factor, btw design (age)	ANOVA	0.058 (age)	12 y, 5 y; 17 a, 5 a

y, Young; m, middle-aged; a, aged; btw, between-factor of the ANOVA; win, within-factor of the ANOVA; *N* depicts the number of animals, or the number of slices for electrophysiological experiments; D1 and D2 refer to day 1 and day 2 of training or testing, respectively; p2/p1, EPSP slope in response to the second pulse divided by the EPSP slope in response to the first pulse.

## Results

### Morbidity

The study began by single housing 17 mice from each age group and ended with tissue harvesting 75 d later. All mice in the young and middle-aged groups survived the 75 d of the study, but three mice from the aged group died during this time period, and three more had to be removed from that group due to morbidity. Thus, as expected, mortality was higher in the aged group when compared to the other groups (χ^2^ = 14.01, *p* = 0.001^a^; Fig. 1B).

### Normal locomotor and rearing activity in an open field, with mild decreases in center activity in aged mice

**Figure 2.**
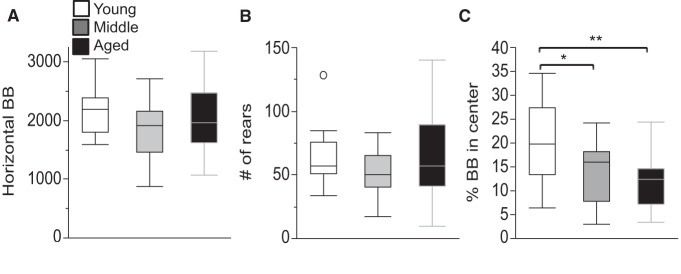
Unchanged locomotor and rearing activity, but reduced percentage of activity in the center of the arena in aged mice. ***A***, ***B***, The total number of horizontal beam breaks (***A***) and rearings (***B***) are shown. No significant differences were detected between any of the age groups in either measure. ***C***, The percentage of beam breaks that were recorded in the center of the locomotor arena is shown. Values are expressed as box plots, as defined in the Data analysis section of Materials and Methods. BB, Beam breaks. *p < 0.05, **p < 0.005.

Age did not affect either horizontal locomotion (Fig. 2A; *p* = 0.27^b^) or vertical rearings (Fig. 2B; *p* = 0.24^c^) during the 15 min recording period in a novel open field. There was, however, a small but statistically significant decrease in the proportion of movement in the center of the open field from ∼20% in young mice to 14% and 12% in middle-aged and older-aged mice, respectively (Fig. 2*C*; significant effect of age by ANOVA; *F*_(2,48)_ = 6.22, *p* = 0.004; *post hoc* comparisons: young vs aged: *p* = 0.004; young vs middle-aged: *p* = 0.027; middle-aged vs aged: *p* = 0.73^d^).

### Normal gross visual function in all age groups

Gross visual function was assessed by scoring the response of the mouse when lowered onto a table surface (visual placing) or when a cotton swab approached the eyes (eyeblink testing). All mice tested (17 young, 17 middle-aged, and 12 aged) showed the expected visual placing responses, with the exception of one aged mouse that consistently failed to show the forepaw extension response as well as the blink response in one eye.

### Impaired Barnes maze performance in aged mice

Spatial learning and memory was evaluated by a Barnes maze procedure. One mouse was excluded from Barnes maze analyses due to visual impairment as assessed in the visual placing and eyeblink tests (see above).

#### Training phase

**Figure 3.**
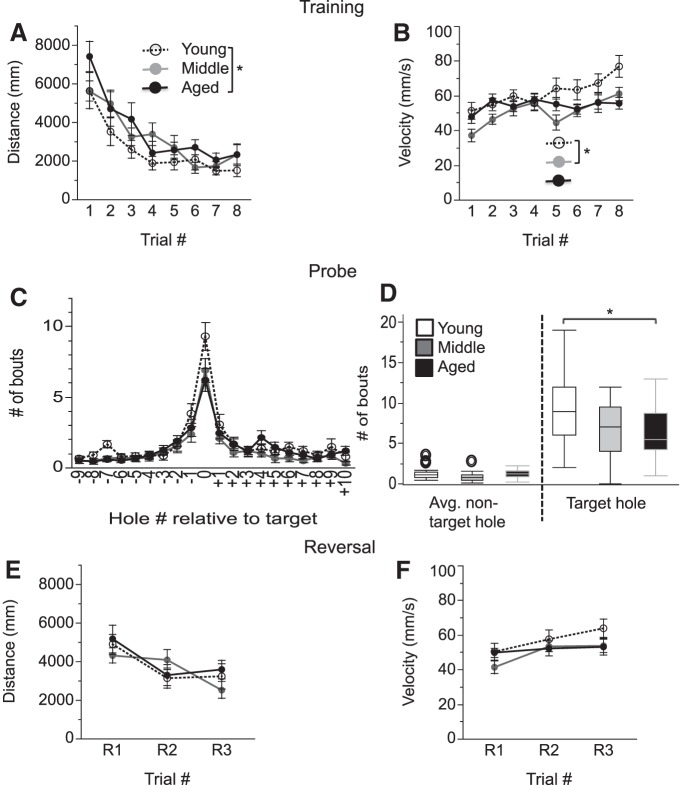
Barnes maze performance is impaired in aged mice. ***A***, ***B***, Training sessions. The distance travelled (***A***) and velocity of movement (***B***) until the mice entered the escape tunnel during training are shown. ***C***, ***D***, Probe trials. ***C*** shows the number of exploration bouts that were recorded at the location of the target hole (defined as position 0) as well as each individual nontarget hole. The absolute value of the “hole number relative to the target” reflects the distance from the target hole. ***D***, The average number of exploration bouts at the nontarget and target holes are shown. ***E***, ***F***, Reversal phase; the distance travelled (***E***) and the velocity of the movement (***F***) during the reversal phase of the task are shown. Values are expressed as the mean ± SEM (***A–C***, ***E***, ***F***) or box plots, as defined in the Data analysis section in Materials and Methods (***D***). *p < 0.05.

While all groups demonstrated learning (improvement with increasing number of trials), age did affect the training process. Specifically, two-way ANOVA of the distance travelled to the escape tunnel revealed main effects of age (*F*_(2,46)_ = 4.38, *p* = 0.018) and trial number (*F*_(7,325)_ = 22.40, *p* < 0.0001), but no interaction of age by trial number (*p* = 0.67). *Post hoc* tests showed that aged mice travelled significantly longer distances when compared to young mice (young vs aged mice: *p* = 0.015; young vs middle-aged mice: *p* = 0.15; middle-aged vs aged mice: *p* = 0.55; Fig. 3A ^e^). ANOVA of the velocity of movement revealed the main effects of age (*F*_(2,47)_ = 3.33, *p* = 0.044; young vs middle-aged: *p* = 0.03; young vs aged: *p* = 0.25; middle-aged vs aged: *p* = 0.64; and trial number: *F*_(7,325)_ = 9.17, *p* < 0.0001) and an interaction effect of age by trial number (*F*_(14,325)_ = 2.26, *p* = 0.006; Fig. 3B ^f^).

#### Probe trial

In order to minimize the possibility that age-dependent changes in velocity of movement affect the probe results, probe trial data analyses were based on the number of exploratory bouts rather than measures that are directly dependent on velocity, such as latency or distance readouts. All age groups showed a strong preference for searching at the correct target hole, although differences between young and aged mice were observed (Fig. 3*C*). ANOVA of the number of bouts spent at target versus nontarget holes revealed the main effects of age (*F*_(2,47)_ = 3.6, *p* = 0.035; *post hoc* tests: young vs aged: *p* = 0.047; young vs middle-aged: *p* = 0.087; middle-aged vs aged: *p* = 0.95) and hole type (target vs nontarget: *F*_(1,47)_ = 164.83, *p* < 0.0001; target > nontarget), and a trend toward an age by hole type interaction (*F*_(2,47)_ = 3.12, *p* = 0.053). Importantly, the number of bouts at the target hole was significantly higher for young relative to aged mice (young vs aged: *p* = 0.043; young vs middle-aged: *p* = 0.13; middle-aged vs aged: *p* = 0.84), but there was no significant difference between any of the age groups at the nontarget holes (young vs aged: *p* = 0.77; young vs middle-aged: *p* = 0.15; middle-aged vs aged: *p* = 0.47; Fig. 3D ^g^). These data indicate that all mice had similar patterns of exploration at nontarget holes, and that the deficits in aged mice relate mostly to targeting the correct hole. In light of the observation that aged mice moved less in the center of the open field (Fig. 2*C*), we wanted to further explore the potential impact of exploratory behaviors on Barnes maze probe trial performance. Movement in the center of the arena during the open field test in aged mice was therefore correlated with the difference between the number of exploratory bouts directed toward the target hole and nontarget holes (number of bouts) in the Barnes maze for aged mice. These measures were significantly correlated (*p* = 0.046, *r* ^2^ = 0.25), suggesting that ∼25% of the variance on either of these measures is accounted for by the other measure, while the majority of the variance (75%) is not^h^.

#### Reversal training

Age did not affect the reversal phase of the task, with all age groups showing improved performance with an increasing number of trials. ANOVA for the distance travelled during the three reversal trials revealed a main effect of trial (*F*_(2,93)_ = 12.53, *p* < 0.0001), but no age-dependent effects (main effect of age: *p* = 0.77; age by trial number interaction: *p* = 0.14; Fig. 3E ^i^). Similarly, ANOVA for the velocity of movement revealed a main effect of trial (*F*_(2,92)_ = 8.21, *p* = 0.0005), but no age-dependent effects (main effect of age: *p* = 0.28, age by trial interaction: *p* = 0.36; Fig. 3F ^j^**)**.

### No clear age-dependent changes in fear conditioning

Fear learning and memory were tested using a classic fear-conditioning paradigm in which an innate freezing response was used as readout for the ability of mice to learn and remember an acoustic cue (cued FC) or a context (contextual FC) that is associated with an electric shock.

#### Training

**Figure 4.**
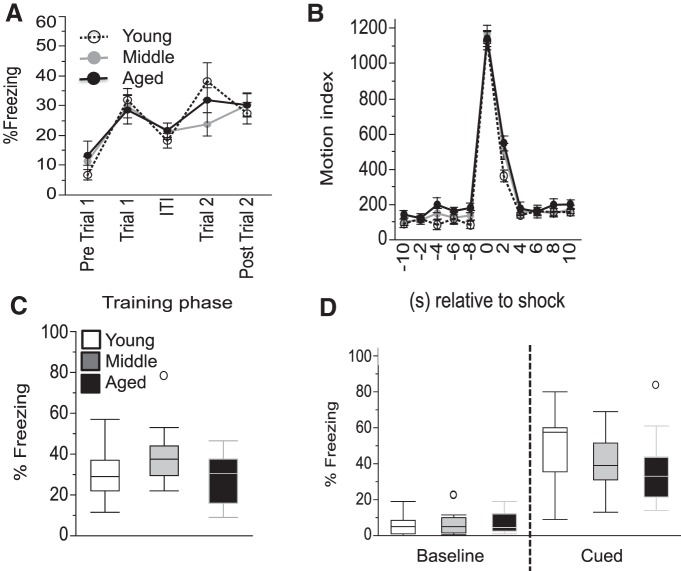
Contextual and cued fear conditioning training and testing are largely unaffected by age. ***A***, The time course of freezing throughout the training is shown. Trial 1 and 2 refer to the first and second pairings of the NS or CS with the UCS. ***B***, The time course of the motor responses in the training phase relative to the footshock is shown with a 2 s resolution using average values from the two trials. ***C***, Percentage of freezing during the contextual FC test is shown. ***D***, Percentage of freezing during the baseline and cued phase of the cued FC test is shown. Values are expressed as the mean ± SEM (***A***, ***B***) or box plots, as defined in the Data analysis section in Materials and Methods (***C***, ***D***). NS, Neutral stimuli.

During training, all groups behaved similarly, regardless of age. Specifically, ANOVA of the percentage of freezing with the factors age and training phase with the levels “pre-UCS” and “post-UCS” revealed only the expected main effect of time (*F*_(1,45)_ = 61.73, *p* < 0.0001; “pre-UCS” < “post UCS”), but no age-dependent effects (main effect of age: *p* = 0.42; age by time interaction: *p* = 0.82; Fig. 4*A* ^k^). Similarly, ANOVA of the motion index with the factors age and time with the levels pre-UCS and “during UCS” revealed only the expected main effect of time (*F*_(1,45)_ = 1207.71, *p* < 0.0001; pre-UCS < during UCS), but no age-related effects (main effect of age: *p* = 0.65; age by time interaction: *p* = 0.67^l^; see Fig. 4*B* for the time course of the response to the UCS). Thus, the baseline motion and the motion response during the shocks were similar between groups, indicating a similar immediate response to the shock.

#### Contextual FC test

There were mild age-dependent differences in freezing in the context test (main effect of age by ANOVA: *F*_(2,45)_ = 3.30, *p* = 0.046) with numerically higher freezing levels for the middle-aged group, but none of the individual age groups were significantly different from each other based on Tukey’s *post hoc* tests (young vs aged: *p* = 0.89; young vs middle-aged: *p* = 0.12; middle-aged vs aged: *p* = 0.057; Fig. 4C ^m^).

#### Cued FC test

**Figure 5.**
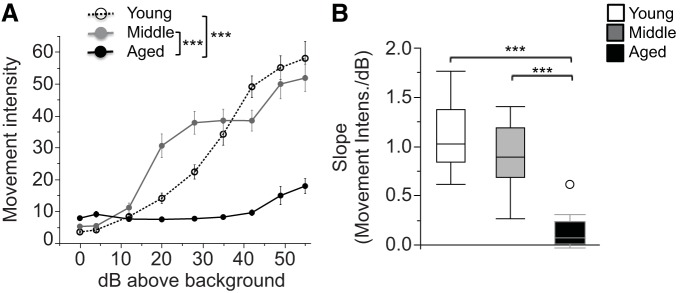
Acoustic startle responses are reduced in aged mice. ***A***, The movement intensity (artificial units) in response to 40 ms pulses of white noise (0–55 dB above background) is shown. ***B***, The slope of the movement intensity over the stimulus intensity (in decibels above background) is shown. Values are expressed as the mean ± SEM (***A***) or box plots, as defined in the Data analysis section in Materials and Methods (***B***). ***p < 0.0001.

The age groups had similar freezing levels when placed in an altered context in the absence of a cue, but there was a trend toward an age effect on freezing when the CS cue was played. A two-way ANOVA of the percentage of freezing with the factors age and time with the factor levels of time before or during the CS was performed. It revealed the expected effect of time (*F*_(1,45)_ = 231.58, *p* < 0.0001) with a significantly higher percentage of freezing during the CS, and an interaction of age by time (*F*_(2,45)_ = 3.78, *p* = 0.030), but no main effect of age (*p* = 0.31). *Post hoc* tests for the percentage of freezing for the time window before the CS revealed no age-dependent effect (young vs aged: *p* = 0.72; young vs middle-aged: 0.73; middle-aged vs aged: 0.99), and only a trend for the time window during the CS for young mice to have more freezing relative to aged mice (*p* = 0.12; young vs middle-aged: *p* = 0.24; middle-aged vs aged: *p* = 0.88; Fig. 4*D* ^n^). It is important to evaluate these data in the context of findings from acoustic startle testing (Fig. 5*A*,*B*) indicating potential hearing deficits in aged mice (see below).

### Pronounced acoustic startle deficits in aged mice

Movement intensities in startle chambers in response to varying degrees of acoustic stimulus intensities were used to screen for basic hearing function. Pronounced age-dependent differences were detected, with reduced responses in aged mice. ANOVA of movement intensity with the factors age and stimulus intensity (in decibels above background) revealed the main effects of age (*F*_(2,45)_ = 34.28, *p* < 0.0001) and stimulus intensity (*F*_(8,360)_ = 123.27, *p* < 0.0001), and an age × stimulus intensity interaction (*F*_(16,360)_ = 23.61, *p* < 0.0001; Fig. 5*A*). *Post hoc* tests showed that both young and middle-aged mice had significantly higher movement intensities when compared with aged mice (young vs aged: *p* < 0.0001; young vs middle-aged: *p* = 0.64; middle-aged vs aged: *p* < 0.0001^°^). To extract the most meaningful data out of any intensity-dependent effects while avoiding multiple-comparison problems with this nine-level factor, slopes of the movement intensity over stimulus intensity were submitted to one-way between-subjects ANOVAs. These analyses revealed a significant age effect (*F*_(2,45)_ = 44.06, *p* < 0.0001; Fig. 5*B*) with aged mice responding significantly less to increases in stimulus intensities as indicated by greater slopes (movement intensity/decibels above background; *post hoc* tests: young vs aged: *p* < 0.0001; young vs middle-aged: *p* = 0.12; middle-aged vs aged: *p* < 0.0001^p^). While this test is not designed specifically to test hearing, it is sensitive to gross deficits in the ability to perceive broadband sounds. Our observation of deficits in aged mice is consistent with previous reports in mice from this strain (Johnson et al., 1997; Francis et al., 2003), indicating that aged C57BL/6 mice are likely to have significant hearing deficits.

### Pronounced active avoidance deficits in aged mice

Associative learning and memory were probed by recording the responses of the animal to explicit acoustic and visual cues (multimodal active avoidance) or primarily visual cues (visual active avoidance) that are presented prior to avoidable electric shocks. To avoid the shock, the mice must move from the compartment where the cue is presented to the opposite compartment.

#### Multimodal AA

**Figure 6.**
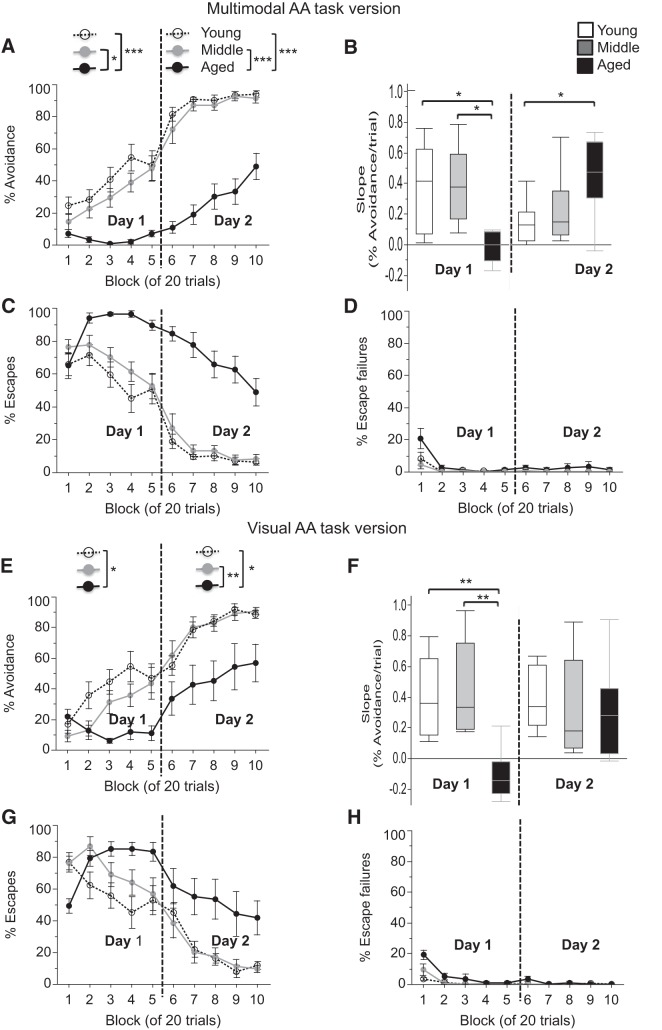
Active avoidance is impaired in aged mice in both multimodal and visual versions of the task. ***A***, ***E***, Percentage of successful avoidance trials is shown in blocks of 20 trials over the 2 day test period for the multimodal (***A***) and visual (***E***) version of the task. ***B***, ***F***, Slopes of the percentage of avoidance per trial are shown for the multimodal (***B***) and visual (***F***) AA task for both training days. ***C***, ***D***, ***G***, ***H***, The percentages of escapes (***C***, ***G***) and escape failures (***D***, ***H***) are shown in blocks of 20 trials over the 2 day test period for the multimodal (***C***, ***D***) and visual (***G***, ***H***) AA task. Values are expressed as the mean ± SEM (***A***, ***C***, ***D***, ***E***, ***G***, ***H***) or box plots, as defined in the Data analysis section in Materials and Methods (***B***, ***F***). *p < 0.05, **p < 0.005, ***p < 0.0001.

The aged group showed substantially reduced avoidance responses relative to the two younger groups (Fig. 6*A*). While young and middle-aged mice steadily increased the number of avoidance responses with training, approaching maximal avoidance by the middle of day 2, aged mice had no appreciable increase in avoidance responses on day 1 and slowly increasing avoidance responses on day 2. Data from each of the 2 training days were analyzed by ANOVA with the factors age and block of trials. For day 1, ANOVA revealed the main effects of age (*F*_(2,21)_ = 17.03, *p* < 0.0001) and block of trials (*F*_(4,84)_ = 14.38, *p* < 0.0001), and an interaction of age × block of trials (*F*_(8,84)_ = 4.59, *p* = 0.0001). *Post hoc* analysis revealed that avoidance was lower in aged mice when compared with young and middle-aged mice on day 1 (young vs aged: *p* < 0.0001; young vs middle-aged: *p* = 0.36; middle-aged vs age: *p* = 0.001^q^). Similar analyses for day 2 again revealed the main effects of age (*F*_(2,84)_ = 69.93, *p* < 0.0001) and a block of 20 trials (*F*_(4,84)_ = 19.38, *p* < 0.0001), and an interaction of age × block of 20 trials (*F*_(4,84)_ = 3.01, *p* = 0.005). *Post hoc* analysis revealed that the percentage of avoidance was lower in aged mice when compared with young and middle-aged mice on day 2 (young vs aged: *p* < 0.0001; young vs middle-aged: *p* = 0.79; middle-aged vs aged: *p* < 0.0001^r^; Fig. 6*A*). An ANOVA of the slopes of the percentage of avoidance over blocks of five trials, an indicator of the learning progress with an increasing number of trials, revealed a main effect of age for day 1 (*F*_(2,21)_ = 8.13, *p* = 0.002), with significantly shallower slopes in aged mice when compared with young and middle-aged mice (*post hoc*: young vs aged: *p* = 0.007; young vs middle-aged: *p* = 0.98; middle-aged vs aged: *p* = 0.005^s^). For day 2, there was also a main effect of age (*F*_(2,21)_ = 4.46, *p* = 0.024), but with significantly steeper slopes in aged mice when compared with young mice (*post hoc*: young vs aged: *p* = 0.023; young vs middle-aged: *p* = 0.72; middle-aged vs aged: *p* = 0.11^t^; Fig. 6*B*). This shift in the magnitude of the slope on day 2 reflects the fact that the young and middle-aged mice have already approached maximum avoidance and have little room to improve, while the older mice have just started learning the task and have a bigger window for improvement on day 2. Overall, these data show that aged mice have pronounced delays in the acquisition of the task when compared with the two younger age groups. It is also important to note that aged mice had a substantially higher percentage of escapes when compared with young and middle-aged mice (Fig. 6*C*) and that the number of escape failures was near zero for all groups at the end of the training period (Fig. 6*D*). These data confirm the aversiveness of the shock and the motivation to avoid or escape it in all groups.

#### Visual AA

While the results of the multimodal AA task suggest a cognitive deficit in the aged mice, the hearing deficits revealed by the acoustic startle testing raise the possibility that an inability to hear the CS could contribute to the poor performance of the aged mice on this task. To investigate this possibility further, an AA task was conducted in which the acoustic salience of the CS was varied by leaving the light cue, but eliminating the tone as an explicit acoustic CS in the task. Once again, age-dependent differences were detected, with reduced avoidance responses in the aged group relative to young mice (Fig. 6*E*). Data from each of the 2 training days were analyzed with an ANOVA with the factors age and block of trials. For day 1, ANOVA revealed the main effects of age (*F*_(2,21)_ = 3.90, *p* = 0.036) and block of trials (*F*_(4,84)_ = 10.77, *p* < 0.0001), and an interaction of age × block of trials (*F*_(8,84)_ = 6.76, *p* < 0.0001). *Post hoc* analysis revealed that the percentage of avoidance was lower in aged mice when compared with young mice on day 1 (*p* = 0.029; young vs middle-aged mice: *p* = 0.30; middle-aged vs aged mice: *p* = 0.35^u^). Similar analyses for day 2 again revealed the main effects of age (*F*_(2,21)_ = 8.17, *p* = 0.002) and block of trials (*F*_(4,84)_ = 21.93, *p* < 0.0001), but no interaction of age × block of trials (*p* = 0.74). *Post hoc* analysis revealed that the percentage of avoidance was lower in aged mice when compared with young and middle-aged mice (young vs aged: *p* = 0.005; young vs middle-aged: *p* = 0.98; middle-aged vs aged: *p* = 0.004^v^; Fig. 6*E*). On day 1, an ANOVA of the slopes of the percentage of avoidance over blocks of five trials, revealed a main effect of age (*F*_(2,21)_ = 9.78, *p* = 0.001). *Post hoc* tests revealed significantly lower slopes in aged mice when compared with young and middle-aged mice (young vs aged: *p* = 0.003; young vs middle-aged: *p* = 0.91; middle-aged vs aged: *p* = 0.001^w^). On day 2, an ANOVA of the slopes of the percentage of avoidance over blocks of five trials did not reveal a main effect of age (*p* = 0.80; Fig. 6F ^x^). In young and middle-aged mice, the percentage of avoidance during the first block on day 2 was numerically lower in the visual AA task when compared with the multimodal AA task (Fig. 6, compare *A*, *E*), which arguably increased the likelihood that further improvement in learning can be detected. As with the multimodal task, these data again show that aged mice have pronounced delays in the acquisition of the task. Aged mice had a substantially higher percentage of escapes when compared with young and middle-aged mice (Fig. 6*G*), and the number of escape failures was near zero for all groups at the end of the training period (Fig. 6*H*), again confirming the aversiveness of the shock and the motivation to avoid and or escape it in all groups.

As with the Barnes maze analyses, we wanted to determine whether age-dependent changes in exploratory behavior (as measured by locomotion in the center of the open field) could affect active avoidance behavior. Data were combined from the multimodal and visual AA tasks and correlations between the percentage of beam breaks in the center of the open field and the slope of the percentage of avoidance by trial from day 1 were calculated in aged mice. There was no significant correlation between these measures (*p* = 0.54, *r* ^2^ = 0.033^y^), representing <4% of the variance and suggesting that the deficits in active avoidance observed in old mice cannot be explained by altered exploratory behavior.

### Visual active avoidance and optokinetic testing in an independent cohort of mice

Visual placing tests in cohorts 1–3 did not suggest any impact of visual functioning on AA behavior. In order to better investigate any potential link between decrements in visual function and AA behavior, we have conducted visual AA testing followed by detailed parametric visual testing based on optokinetic responses in a separate, large cohort of mice (cohort 4) and conducted correlational analyses between key measures from the two behaviors.

#### Visual AA (cohort 4)

**Figure 7.**
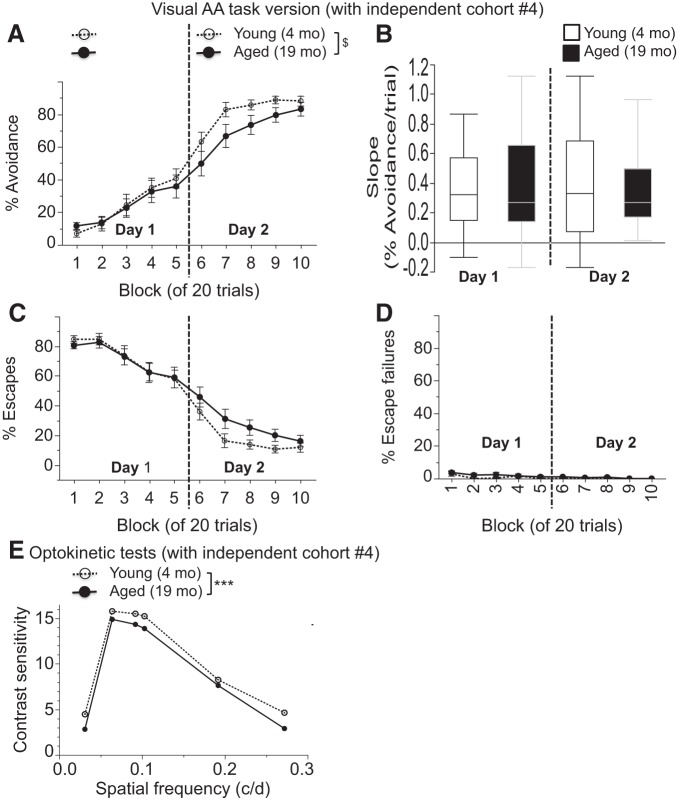
***A–E***, Active avoidance (***A–D***) and optokinetic testing (***E***) in an independent cohort. Visual active avoidance is mildly impaired in 19-month-old mice. ***A***, The percentage of successful active avoidance trials is shown in blocks of 20 trials over the 2 day test period for the visual version of the task. ***B***, Slopes of the percentage of avoidance per trial are shown for both AA training days. ***C***, ***D***, The percentages of escapes (***C***) and escape failures (***D***) are shown in blocks of 20 trials over the 2 day test period. ***E***, Contrast sensitivity from optokinetic tests is shown across varying spatial frequencies (in c/d). Values are expressed as the mean ± SEM. SEMs are not visible in ***E*** since they range from only 0.014 to 0.092 across age groups and spatial frequencies. ^$^*p* < 0.1, ***p < 0.0001.

Mice 23 to 25 months old were not available at the time of testing, so we used a large group of 19-month-old mice. These 19-month-old mice appeared to have an intermediate AA phenotype when compared to the younger and older mice tested in cohorts 1–3 (compare Fig. 6*E*). Specifically, on day 1, 19-month-old mice performed similar to 4-month-old mice, with ANOVA showing a block of trial effects (*F*_(4,132)_ = 29.56, *p* < 0.0001), but no effect of age (*F*_(1,33)_ = 0.01, *p* = 0.92) or interaction of age × block of trials (*F*_(1,132)_ = 0.61, *p* = 0.65^z^). Similar analyses for day 2 revealed a trend toward a main effect of age (*F*_(1,33)_ = 2.88, *p* = 0.099) and a block of trial effects (*F*_(4,132)_ = 39.22, *p* < 0.0001), but no interaction of age × block of trials (*F*_(4,132)_ = 1.30, *p* = 0.27^aa^; Fig. 7*E*). Correspondingly, ANOVA of the slopes of the percentage of avoidance over blocks of five trials did not reveal a main effect of age for day 1 (*F*_(1,33)_ = 0.745, *p* = 0.39^ab^) or day 2 (*F*_(1,33)_ = 2.14, *p* = 0.15^ac^). As before (Fig. 6*H*), the number of escape failures was near zero for all groups at the end of the training period (Fig. 7*D*), again confirming the aversiveness of the shock and the motivation to avoid and or escape it in both age groups.

#### Optokinetic testing (cohort 4)

When vision was assessed by optokinetic testing, 19-month-old mice had mild yet significant decrements in threshold measures of spatial frequency and contrast sensitivity. 19 and 4-month-old mice had an average spatial frequency threshold across clockwise and counter-clockwise rotations of the visual stimuli of 0.329 and 0.386, respectively, with SEM values for each age group of ∼0.0007. ANOVA of the spatial frequency threshold revealed a small, yet highly significant effect of age (*F*_(1,34)_ = 3350.13, *p* < 0.0001^ad^). Similarly, ANOVA of contrast sensitivity data revealed highly significant main effects of age (*F*_(1,34)_ = 612.49, *p* < 0.0001) and spatial frequency (*F*_(5,170)_ = 25,853.44, *p* < 0.0001), and an interaction effect of age by spatial frequency (*F*_(5,170)_ = 38.58, *p* < 0.0001; Fig. 7*E*). Contrast sensitivity displayed the expected inverted U-shaped curve, with peak values at a spatial frequency of 0.064 c/d for both age groups. While the age effect appeared largest at 0.031 c/d and smallest at 0.192 c/d, clearly, these relative differences were small and age differences were all at the *p* < 0.0001 level for each of the spatial frequencies tested. SEM values ranged from only 0.014 to 0.092 across age groups and spatial frequencies^ae^.

These data from cohort 4 now allow for correlational analyses between AA performance and visual function in aged mice, in analogy to the correlational analyses that were conducted between other measures for cohorts 1–3. First, the spatial frequency threshold from each aged mouse was correlated with AA slopes across blocks of five trials for each of the 2 d of AA testing. For day 1, the *r* ^2^ value was 0.048 at *p* = 0.36^af^, and for day 2, the *r* ^2^ value was 0.002 at *p* = 0.85^ag^. Similar analyses between the peak contrast sensitivity of each aged mouse at a spatial frequency of 0.064 c/d with the AA slopes across blocks of five trials from both testing days revealed an *r* ^2^ value of 0.005 at *p* = 0.77^ah^ for day 1 and an *r* ^2^ value of 0.023 at *p* = 0.54^ai^ for day 2. Thus, in none of these cases was there a significant correlation between highly sensitive parametric visual data and active avoidance data in aged mice. No more than 5% of the variance of active avoidance measures could be explained by any of these visual data. Hence, we could not detect any indication that age-related changes in visual function could explain the impaired active avoidance behavior of aged mice.

### Increased gliosis in the hippocampal formation in aged mice

**Figure 8.**
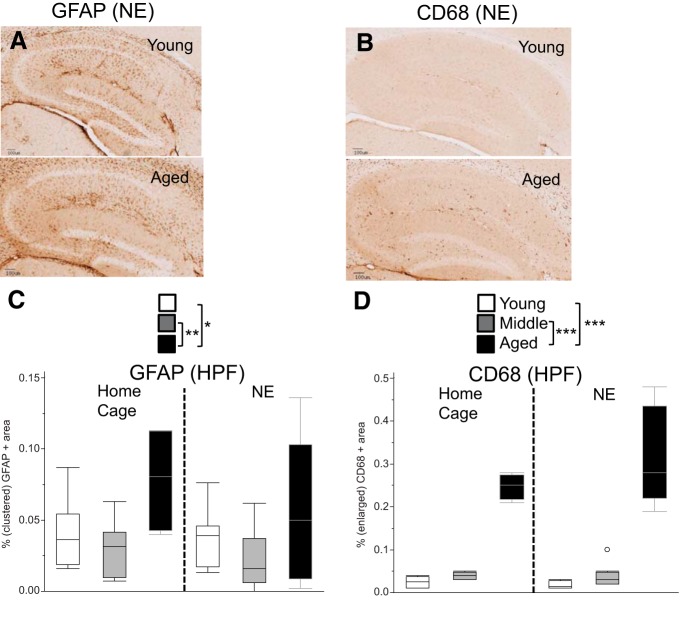
Markers of gliosis (GFAP, CD68) are increased in aged mice. ***A***, ***B***, Examples of brain slices showing areas of GFAP-positive (***A***) and CD68-positive (***B***) staining in the HPF are shown from the NE condition. ***C***, The percentage of the total tissue area that had clustered GFAP-positive staining is shown. ***D***, The percentage of the total tissue area from the HPF that had enlarged CD68-positive staining is shown. Values are expressed as box plots, as defined in the Data analysis section in Materials and Methods. *p < 0.05, **p < 0.005, ***p < 0.0001.

To determine whether age-related changes in gliosis could be involved in some of the deficits observed in aged mice, we stained tissue of the HPF with markers for GFAP (astrogliosis) and CD68 (activated microglia). Since a variation of the environmental conditions (novel vs homecage condition) was introduced in the 2 h prior to tissue harvesting for the purpose of the c-Fos experiment (see below), gliosis data are also analyzed with two-factorial ANOVAs using the factors age and environment. By measuring the percentage of tissue area that showed clustered GFAP immunoreactivity, we observed an increase in GFAP staining in the HPF of aged mice. An ANOVA revealed a main effect of age (*F*_(2,36)_ = 6.23, *p* = 0.005), but no main effect of environment (novel vs homecage environment, *p* = 0.27) and no age × environment interaction (*p* = 0.71; Fig. 8*A*,*C*). *Post hoc* tests revealed that GFAP staining was increased in aged mice relative to young and middle-aged mice with relatively larger differences between middle-aged and aged mice when compared with young and aged mice (young vs aged: *p* = 0.049; young vs middle-aged: *p* = 0.45; middle-aged vs aged: *p* = 0.003^aj^). Increased staining could be caused by a change in the number or morphology or GFAP-positive cells, or simply an increase in the expression level of GFAP per cell. In order to better understand the nature of the cellular changes, we evaluated the density, size, and shape of GFAP-positive clusters. First, the average density of GFAP clusters was increased with age, with ANOVA revealing a main effect of age (*F*_(2,36)_ = 5.75, *p* = 0.007) and a trend toward an environment effect (*F*_(1,36)_ = 3.63, *p* = 0.065), but no age × environment effect (*F*_(2,36)_ = 0.41, *p* = 0.67). *Post hoc* tests revealed that GFAP cluster density was increased in aged mice relative to middle-aged mice and there was a trend in the same direction for the comparison of aged versus young mice (young vs aged: *p* = 0.065; young vs middle-aged: *p* = 0.45; middle-aged vs aged: *p* = 0.005^ak^). ANOVA of the average GFAP cluster size did not reveal any age-dependent effect (main effect of age: *F*_(2,35)_ = 1.35, *p* = 0.27; main effect of environment: *F*_(1,35)_ = 2.73, *p* = 0.11; age × environment: *F*_(2,35)_ = 1.65, *p* = 0.21^al^), neither did the ANOVA on the corresponding shape factor data (main effect of age: *F*_(2,35)_ = 1.07, *p* = 0.35; main effect of environment: *F*_(1,35)_ = 0.52, *p* = 0.47; age × environment interaction: *F*_(2,35)_ = 0.98, *p* = 0.39^am^). Thus, age-dependent increases in the percentage of the GFAP-positive clustered area appear to be mainly linked to age-dependent increases in GFAP-positive cluster density. Quantification of the percentage of CD68-positive tissue similarly showed an increase in microglial activation in the aged HPF. ANOVA of the percentage of the CD68-positive tissue area revealed a main effect of age (*F*_(2,35)_ = 122.79, *p* < 0.0001), but only trends were observed for environment (*F*_(1,35)_ = 2.38, *p* = 0.132) as well as age × environment interaction (*F*_(2,35)_ = 2.60, *p* = 0.088; Fig. 8*B*,*D*). *Post hoc* tests revealed that CD68 staining was significantly enhanced in aged mice relative to young and middle-aged mice (young vs aged: *p* < 0.0001; young vs middle-aged: *p* = 0.461; middle-aged vs aged: *p* < 0.0001^an^). Further analysis revealed an increase in the density of CD68-positive cells, with ANOVA demonstrating a main effect of age (*F*_(2,35)_ = 136.83, *p* < 0.0001), but no main effect of environment (*F*_(1,35)_ = 1.97, *p* = 0.17) and no age × environment effect (*F*_(2,35)_ = 1.79, *p* = 0.18). *Post hoc* tests revealed that CD68-positive object density was increased in aged mice relative to young and middle aged-mice (young vs aged: *p* < 0.0001; middle-aged vs aged: *p* < 0.0001; young vs middle-aged: *p* = 0.13^ao^). The size of CD68-positive objects was also increased with age: ANOVA revealed a main effect of age (*F*_(2,35)_ = 31.54, *p* < 0.0001), but no main effect of environment (*F*_(1,35)_ = 2.55, *p* = 0.12) and no age × environment effect (*F*_(2,35)_ = 0.29, *p* = 0.75). *Post hoc* tests revealed that CD68-positive object size was increased in aged mice relative to young and middle aged-mice (young vs aged: *p* < 0.0001; middle-aged vs aged: *p* < 0.0001; young vs middle-aged: *p* = 0.79^ap^). Further analysis also indicated that the shape of CD68-positive clusters changed with age: ANOVA of the average shape factor of CD68-positive objects revealed a main effect of age (*F*_(2,35)_ = 35.26, *p* < 0.0001), but no main effect of environment (*F*_(1,28)_ = 0.28, *p* = 0.60) and no age × environment effect (*F*_(2,35)_ = 1.47, *p* = 0.24). *Post hoc* tests revealed that the CD68-positive shape factor was increased in aged and middle-aged mice relative to young mice (young vs aged: *p* < 0.0001; young vs middle-aged: *p* < 0.0001; middle-aged vs aged: *p* = 0.47^aq^), suggesting that CD68-positive stained areas in aged and middle-aged mice are less elongated, but rounder in shape when compared with young mice. The age-dependent changes in the density, size, and shape of the CD68-positive objects are consistent with increased microglial activation.

In order to determine whether age-dependent changes in active avoidance behavior are tied to age-dependent changes in gliosis, the slope of the percentage of the AA across blocks of five trials for day 1 was correlated with the percentage of the ROI from the HPF that was stained positive for GFAP and CD68 in aged mice. The *R* ^2^ value for GFAP was 0.004 with a *p* value of 0.87^ar^, and the *r* ^2^ values for CD68 was 0.002 at a *p* value of 0.90^as^. Although the power of this analysis (*n* = 9–10) is too low to conclusively rule out a relationship between gliosis and age-related changes in active avoidance behavior, these data offer little evidence of a direct link between these measures of gliosis and behavior in the aged mice.

### Neuronal physiology

#### Reduced c-Fos expression in the dentate gyrus of aged mice

**Figure 9.**
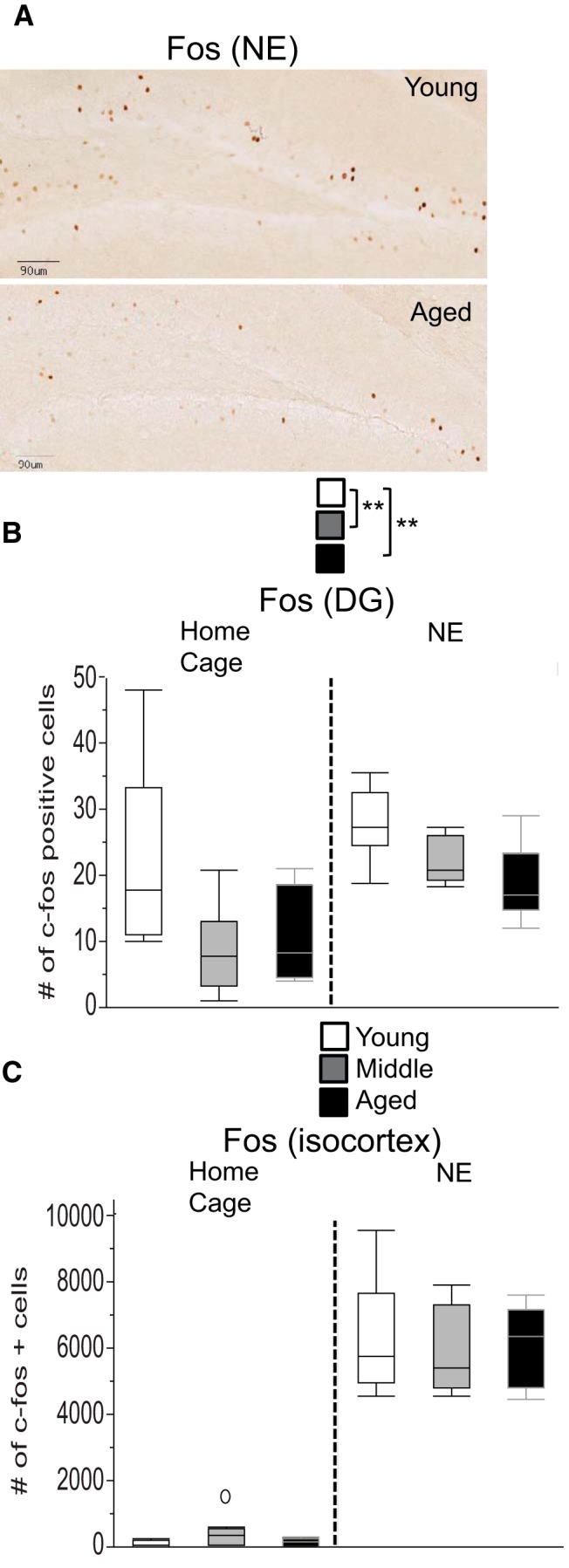
c-Fos expression is reduced in the dentate gyrus, but not in the isocortex, of aged mice. ***A***, Examples of brain slices showing the number of c-Fos-positive cells in the DG are shown from the NE condition. ***B***, Aged mice had reduced c-Fos staining in the DG across environments when compared with young and middle-aged mice. ***C***, In the isocortex, no age-dependent effects were observed. Values are expressed as box plots, as defined in the Data analysis section in Materials and Methods. **p < 0.005.

In order to investigate the neural processes underlying the changes in response to novelty and learning that we observed in aged mice, we exposed mice from each age group to either their standard home cage, or a novel environment for 2 h and then examined the expression of c-Fos, a marker of neuronal activity, in DG and isocortex. In general, young mice had more c-Fos-positive cells in DG but not isocortex compared with older mice, and exposure to a novel environment increased c-Fos-positive cells in DG and isocortex in all age groups. ANOVA of the number of c-Fos-positive cells revealed the main effects of age in the DG (*F*_(2,38)_ = 9.09, *p* = 0.001^at^), but not in the isocortex (*p* = 0.98^au^), and the effects of environment in both DG (*F*_(1,38)_ = 14.11, *p* = 0.001^at^) and isocortex (*F*_(1,38)_ = 296.71, p <0.0001^au^), but no interaction of age with environment for either DG (*p* = 0.30^at^) or isocortex (*p* = 0.56^au^; Fig. 9*A*). *Post hoc* tests revealed a significantly lower number of DG c-Fos-positive cells in aged and middle-aged mice when compared with young mice (young vs aged: *p* = 0.004; young vs middle-aged: *p* = 0.002; middle-aged vs aged: *p* = 0.97^at^; Fig. 9*B*). As expected, the number of c-Fos-positive cells was also significantly higher in the NE condition when compared with the home cage condition in DG (*p* = 0.001^at^; Fig. 9*B*) and isocortex (*p* < 0.0001^au^; Fig. 9*C*). As with the Barnes maze and AA analyses, we wanted to determine whether age-dependent changes in exploratory behavior (as measured by locomotion in the center of the open field) could affect c-Fos induction in the DG. Correlations between the percentage of beam breaks in the center of the open field and the number of c-Fos-positive cells in the DG were calculated in aged mice. There was no significant correlation between these measures (*p* = 0.47, *r* ^2^ = 0.068^av^), indicating that <7% of the variance is shared between these measures. With the limitation that arises from the sample size of only 10 aged mice, for this analysis these data suggest that these two measures are largely independent in aged mice.

#### Altered electrophysiological measures of input–output relationship and paired-pulse facilitation, with nonsignificant trends toward impairments of LTP in aged mice

**Figure 10.**
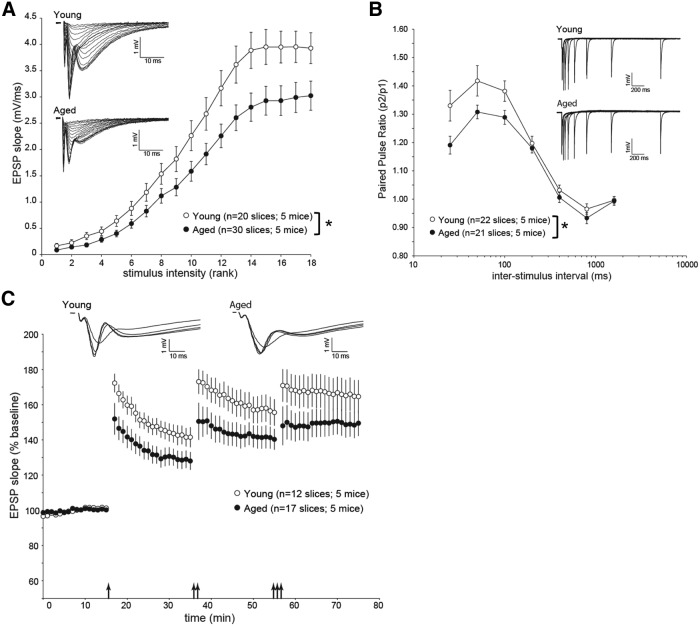
Age-dependent changes in electrophysiological measures of synaptic function in hippocampal area CA1. ***A***, Field EPSPs measured in CA1 in response to stimulation of the Schaffer collateral pathway were reduced in aged mice. Initial EPSP slope is shown in response to increasing stimulation intensity (up to 1 mA, logarithmically spaced increments). Example traces from young and aged mice are shown in the inset. ***B***, The PPR [EPSP slope in response to the second pulse (p2) divided by the EPSP slope in response to the first pulse (p1)] is shown as a function of interstimulus interval. Example traces from young and aged mice are shown in the inset. PPR values >1 reflect PPF. ***C***, The EPSP slope normalized to baseline is shown during the course of the experiment. LTP was induced using TBS. The number of arrows corresponds to the number of TBS bouts. Example traces from young and aged mice are shown in the inset. Values are expressed as the mean ± SEM. PPF, Paired-pulse facilitation. *p < 0.05.

Synaptic physiology was characterized in hippocampal slices from a separate cohort of young and aged mice using field recordings of CA1 pyramidal neuron EPSPs in response to Schaffer collateral stimulation. There was the expected effect of stimulus intensity across all ages (ANOVA main effect of stimulus intensity: *F*_(17,816)_ = 205.40, *p* < 0.0001). Most importantly, aged mice had significantly reduced synaptic strength, as measured by the relationship of stimulus strength to EPSP magnitude (input–output relationship), with significantly lower EPSP slopes in brain slices from aged mice compared with young mice (ANOVA main effect of age: *F*_(1,48)_ = 5.71, *p* = 0.021). This difference between young and aged mice increased with increasing stimulus intensities (ANOVA age × stimulus intensity interaction effect: *F*_(17,816)_ = 407, *p* < 0.0001; Fig. 10A ^aw^). We next examined the response to paired-pulse stimulation, which resulted in facilitation at shorter ISI. The magnitude of such paired-pulse facilitation, as measured by the PPR, is generally believed to be inversely proportionate to presynaptic release probability. ANOVA of the PPR revealed a main effect of age (*F*_(1,50)_ = 5.45, *p* = 0.024) with overall lower PPR in brain slices from aged mice, and a main effect of ISI (*F*_(6,300)_ = 111.05, *p* < 0.0001; Fig. 10*B*). There was an interaction effect of age × ISI (*F*_(6,300)_ = 2.67, *p* = 0.015) with greater differences between brain slices from aged and young mice for shorter ISIs when compared with long ISIs^ax^. The reduced PPR (Fig. 10*B*) in aged mice is consistent with elevated basal presynaptic release probability. Given this indication of enhanced presynaptic release probability, the reduced total synaptic strength seen in the input–output relationship likely reflects either fewer synapses or reduced postsynaptic function in the aged mice. Next, we examined synaptic plasticity by measuring long-term potentiation of synaptic strength, a form of plasticity that is critical for learning and memory. As age-dependent effects may depend on stimulus intensity, we examined LTP in aged mice using increasingly strong induction protocols of one, two, or three bouts of TBS applied sequentially within the same experiment. ANOVA of the EPSP slope did not reveal a significant main effect of age (*F*_(1,27)_ = 2.78, *p* = 0.11) even though EPSP slopes were numerically lower in brain slices from aged mice for all stimulus levels. There was a main effect of the number of TBSs (*F*_(2,54)_ = 43.62, *p* < 0.0001) with greater EPSP slopes for increasing number of TBS, but no interaction effect of age × number of TBS (*p* = 0.88; Fig. 10C ^ay^). Given the relatively small magnitude of the numerical reduction and the not significant LTP reduction, and the observed variability, these results suggest that future studies will need large numbers of recordings to potentially detect LTP impairments in aged mice with high power and low α values. At the same time, it should be noted that an exploratory ANOVA of the present data that was based on LTP responses to one TBS pulse yielded only nearly significant age effects at the present sample size (*F*_(1,27)_ = 3.92, *p* = 0.058^az^), indicating the possibility for improved assay sensitivity to detect age effects if protocols are geared toward weak LTP induction.

## Discussion

We report age-dependent changes in behavior, neuronal activity, synaptic function, and gliosis in C57BL/6 mice. In addition to cognitive tests, we assessed locomotor and sensory functions and adjusted behavioral protocols to minimize the effects of these potential confounds on cognitive measures. Activity in the center of the locomotor arena was reduced, but horizontal locomotion and rearing activity were normal. Startle responsiveness to acoustic stimuli was strongly reduced, while visual placing tests were largely similar between age groups. Aged mice had markedly delayed acquisition of multimodal and visual active avoidance behavior, mild reductions in Barnes maze performance, yet minimal changes in fear conditioning and visual function. Gliosis was increased in aged mice when compared with young mice. In addition, c-Fos induction in the DG of aged mice was reduced, indicating reduced neural activity. This corresponds well with electrophysiological observations of impaired I–O relationship and PPR.

Tests designed to assess cognition in rodents generally require a coordinated motor response to specific environmental cues. Therefore, correct interpretation of these tests requires that sensorimotor abilities be carefully evaluated as well. In our tests of sensorimotor function, we noted a mild decrease in the percentage of activity occurring in the center of an open field for old mice. We confirmed that all but one mouse, regardless of age, had largely normal gross visual ability, as measured by eyeblink and visual placing responses. However, the oldest mice exhibited a reduction in acoustic startle response that was consistent with profound hearing loss in C57BL/6 mice (Li and Borg, 1991; Ouagazzal et al., 2006; Ison et al., 2007; Young et al., 2010; Martin del Campo et al., 2012). We took several approaches to mitigate the effects of these sensorimotor alterations on cognitive measures. First, the individual aged mouse that lacked eyeblink and visual placing responses on one side was simply excluded from analysis in the Barnes maze.

Second, highly sensitive optokinetic testing measures were used to map visual function in detail in a separate cohort of mice. The spatial frequency threshold and contrast sensitivity were mildly attenuated in 19- versus 4-month-old mice; however, visual function was generally well preserved across a range of spatial frequencies and contrasts. This mild loss of visual sensitivity is unlikely to impact performance on a task like AA, where the onset of the cue is a large change in light intensity in an otherwise dark chamber. Third, the findings of a likely hearing loss in C57BL/6 mice suggest that caution is warranted when putative cognitive tests critically rely on acoustic cues, as for instance in FC and multimodal AA. To address this concern, we developed a visual AA version that does not rely on explicit acoustic stimuli, thereby demonstrating the likely independence of the age-dependent decrease in AA performance from concurrent changes in hearing. The potential effect of an altered pattern of exploration, as evidenced by reduced activity in the center of the open field in aged mice, was more difficult to adjust for, since many tests, including the Barnes maze, active avoidance, and novelty-induced c-Fos could be confounded by alterations in anxiety, fear, or exploration. We used a statistical correlation approach to determine how much of the variance in the dependent measures of those tasks could be explained by the percentage of activity that occurred in the center of the open field. We determined that only 25% of the Barnes maze variance could be explained by the open field center exploration. For active avoidance and c-Fos expression, center exploration did not significantly contribute to the variance. Together, most of the variance in these tasks must be explained by aged-related factors other than the observed alterations in open field exploration.

Barnes maze testing (Barnes, 1979) was used to probe spatial memory, a function that is affected in Alzheimer’s disease and normal aging (Moffat and Resnick, 2002; Lithfous et al., 2013). All age groups learned to find the escape hole faster after covering less distance, and demonstrated a pronounced preference for the target hole when compared with the nontarget holes during probe trial testing. Aged mice showed a reduced preference for the target hole, with no significant change in the total exploration of nontarget holes. This suggests that exploratory drive and motivation in the Barnes maze task are similar for all ages, and that the reduced exploration of the target hole observed in aged mice is specific and related to a learning deficit. These findings are consistent with other reports of impaired spatial learning and memory in aged versus young C57BL/6 mice (Bach et al., 1999; King and Arendash, 2002) and are in line with earlier studies in rats (Barnes, 1979). However, the magnitude of effects observed here is relatively small, and potentially partially confounded by the altered exploratory pattern that we observed in aged mice. This suggests that this test has limited sensitivity and specificity for the detection of age-dependent cognitive effects. Similarly, no measures of contextual or cued FC showed robust differences between age groups. This relative lack of age sensitivity is in line with previous reports (Kennard and Woodruff-Pak, 2011). In addition, hearing deficits in aged C57BL/6 mice may confound standard cued FC tasks in aged mice. The age sensitivity of FC can be improved by using trace FC paradigms with long retention phases or by replacing acoustic cues with visual cues (Newton et al., 2004). However, visual FC produces less robust cued freezing responses, and trace FC interrogates a different form of learning. These findings limit the suitability of simple FC tests for future aging studies.

In contrast, clear and consistent age-dependent effects were observed in both multimodal and visual AA tests in young and middle-aged mice relative to aged mice. Interestingly, 19-month-old mice had only a mild AA deficit relative to 4-month-old mice, which is consistent with a gradual development of AA deficits with increasing age. AA behavior reflects a form of associative learning and memory with both elements of classic and instrumental conditioning (Sparkman et al., 2005). Classic conditioning, in particular eyeblink conditioning, is sensitive to age effects in humans (Solomon et al., 1991; Durkin et al., 1993; Woodruff-Pak, 2001), and possibly for the differentiation between likely dementia and normal aging (Solomon et al., 1995; Woodruff-Pak et al., 1996b; Woodruff-Pak et al., 1996a). Hence, these basic forms of learning may be suited for the detection of learning and memory deficits linked to aging and AD-like phenotypes in mammals, including humans. The multimodal AA protocol that was used in the present study led to a rapid acquisition of AA within the first of 2 training days in young C57BL/6 mice (Foldi et al., 2011**).** This is in contrast to several earlier AA protocols that often required several training sessions to achieve similar levels of avoidance in young C57BL/6 mice (Oliverio et al., 1973; Clark et al., 2003; Sparkman et al., 2005). Age-related differences were observed on both training days, which is consistent with an earlier study (Carrié et al., 1999) in the OF1 mouse strain where age-dependent AA deficits developed over a 5 d training period. Similar age deficits were observed in a primarily visual version of the task, indicating that hearing deficits in aged mice are unlikely to account for the observed AA deficits. Altered pain responsiveness is also unlikely to explain the observed effects, given that the response to footshock during FC training did not differ between age groups and the percentage of escape failures was near 0 for all age groups at the end of AA training. One noncognitive explanation that we did not directly investigate is the possibility that aged mice may be more sensitive to stress and that the stress caused by the procedure interfered with the ability of aged mice to perform the task. While beyond the scope of the present investigation, this is a testable hypothesis that could be explored by analyzing whether the corticosterone levels induced by during AA training are linked to performance in this task. Together, AA may represent a reliable behavioral measure for the detection of age-dependent cognitive effects, and may therefore be useful in identifying potential medications to improve age-related cognitive deficits or to test biological pathways that are linked to age-related cognitive decline.

Interestingly, some of the challenges of functional testing in aged mice are also a challenge for neuropsychological testing in aging humans. Age-related cognitive and sensory changes occur concurrently, both contributing to human functional performance (Wood et al., 2005; Glisky, 2007). Human tests that are less dependent on intact sensory and motor function are being developed (Geldmacher et al., 2012). Further, it has been argued that the border between sensory and cognitive processes is difficult to define and that age-related changes in both domains may be the result of “shared” changes in the brain. This concept (Baltes and Lindenberger, 1997; Murray and Bussey, 1999; Glisky, 2007; Burke et al., 2012) has implications for the design of both clinical and preclinical studies. For example, the sensitivity of trials of potential therapeutics to improve or prevent age-dependent changes in the brain may be improved by applying multivariate statistical analyses that include both cognitive and sensory measures as dependent variables.

Microglia and astrocytes have diverse roles in the nervous system, ranging from immune defense to supporting tissue integrity, neurotransmission, and the metabolic environment of the nervous system. Microglia and astrocyte reactivity appears to increase with age. While the detailed time course and functional consequences of these changes are still debated, it has been widely argued that gliosis is linked to cognitive decline in normal aging and age-related brain disease (Barrientos et al., 2015; Rodríguez-Arellano et al., 2015). For example, GFAP and CD68 were significantly enhanced in the cortex of AD patients relative to control subjects, and tended to increase with age in control subjects (Wyss-Coray, 2006). Further, age-dependent increases in GFAP staining have been observed in human hippocampal samples (Takahashi et al., 2006). Thus, in order to investigate cellular substrates associated with the cognitive deficits we observed in aged mice, we also examined gliosis in the HPF. Indeed, both GFAP and CD68 were clearly increased in aged mice. Similarly, age-dependent increases in gliosis have been detected in a range of mouse models of AD (Oakley et al., 2006; Wright et al., 2013; Cheng et al., 2014), sometimes prior to the onset of plaque deposition. This indicates the potential for gliosis-related early disease markers for AD (Wright et al., 2013). HPF CD68 staining has been shown to negatively correlate with cognitive performance in aged rats (Farso et al., 2013). While it is still not clear whether such changes in gliosis trigger or accelerate age-dependent cognitive decline, or if they merely represent epiphenomena of the decline, robust models of such changes are needed to precisely answer these questions. The effects reported in the present study were detected with automated, intensity- and size-dependent, threshold-based algorithms that, in contrast to manual counting methods, do not specifically quantify the absolute number, size, or morphology of GFAP- or CD68-positive individual cells. Therefore, we analyzed other parameters, like density and shape factor of clusters, to obtain some morphological information. Our data suggest that it is primarily the density of GFAP-positive clusters that changes with age, rather than their size or shape. CD68-positive objects, on the other hand, showed evidence of age-dependent alterations in density, size, and shape. This is consistent with the proliferation and morphological changes expected when microglia become activated. Correlational analyses, however, did not reveal a major link between either GFAP- or CD68-positive areas and AA performance in aged mice, suggesting that, at least at the time of these analyses, these measures are largely independent. Thus, our findings demonstrate that robust changes in GFAP and CD68 staining can be efficiently detected in this mouse model. Together, the observed effects may have some important translational value for human aging, may serve as a basis to link the underlying basic biology to the observed cognitive measures, and may provide a basis for testing the hypothesis that altering gliosis in the brain can ameliorate age-dependent cognitive decline in rodents.

If the behavioral changes we observed truly reflect age-related cognitive deficits, we would expect that there would be concurrent changes in neural activity and synaptic function. We used c-Fos expression and induction in the DG as a surrogate marker for neuronal activity in response to novel stimuli. We detected age-related effects on c-Fos expression in the DG, but not in the isocortex. This is notable because much of the cortical c-Fos induction is due to activation of sensory and motor cortical regions. So in analogy to the behavioral experiments, the cortical c-Fos expression can serve as a control for normal sensory and motor responses. Functionally, the DG is important for associative and spatial learning and memory, as well as for the response to novelty. Age-dependent effects in the DG, however, were present in both the NE and the home-cage conditions, suggesting that age-related differences in brain activity not only occur during specific experimental challenges, but also are present under baseline conditions in aged mice. This is consistent with previous studies in rats (Lee et al., 1998). In mice, age-dependent reductions in c-Fos immunoreactivity in the DG were observed in the DG of 6- to 7-month-old mice relative to 4- to 5-month-old mice of the J20 model of Alzheimer’s disease. c-Fos levels were also dramatically lower in J20 mice at both ages when compared with nontransgenic mice. In 6- to 7-month-old J20 mice, c-Fos immunoreactivity in the DG correlated with functional impairment in the Morris water maze (Palop et al., 2003, 2005). It appears that c-Fos expression in the DG may represent an age-sensitive and possibly robust measure, given that age effects were observed across two conditions with markedly distinct levels of novelty.

A more direct measure of age-dependent changes in neuronal activity and plasticity comes from electrophysiological studies in hippocampal slice preparations. Brain slices from aged mice had significantly reduced synaptic strength, as measured by the I–O relationship. Age-dependent differences were most pronounced for higher stimulus intensities, which is in line with studies in rats (Norris et al., 1998; Kumar and Foster, 2013). Next, we assessed presynaptic release probability using paired-pulse stimulation protocols. Paired-pulse stimulation typically results in PPFs at shorter ISIs, a phenomenon considered to be an inverse correlate of presynaptic release probability (Schulz et al., 1994; Asztely et al., 1996). Brain slices from aged mice had significantly reduced PPF. Age-dependent differences were most pronounced for short ISIs, which is consistent with increased release probability. While multiple interpretations could exist for reduced total synaptic strength accompanied by increased release probability, one potential explanation is that increased release probability occurs as a compensatory response to fewer synaptic inputs. This is consistent with data from the PS2APP mouse model of AD, which exhibits synapse loss (as measured by decreased postsynaptic spine density) together with reduced PPF, indicating that increased release probability may occur as a compensation for synapse loss (Hanson et al., 2014).

To test for a correlate of impaired learning and memory in aged mice, we measured hippocampal LTP (Lynch, 2004; Miyamoto, 2006; Stuchlik, 2014). Our results only show numerically smaller but nonsignificant EPSP slopes for aged mice when compared with young mice for each stimulus condition. Studies of the effects of aging on NMDA-dependent hippocampal LTP have produced different results, with some reports showing age-dependent deficits, and others not (Deupree et al., 1993; Barnes et al., 1996; Norris et al., 1996; Rosenzweig et al., 1997; Bach et al., 1999; Rosenzweig and Barnes, 2003). This may in part be due to differences in the induction and recording protocols used. First, the length of the LTP recording likely is critical. Age-dependent effects may be more likely to manifest when recordings of >1 h are obtained (Rosenzweig and Barnes, 2003). Second, since stimulus intensity may be critical, we explored different induction protocol strengths (one, two, or three TBS bouts) to best detect the potential effects of age. Indeed, while interactions between age and the number of TBS bouts used to induce LTP were not significant, age-dependent differences in LTP expression were largest with fewer TBS bouts and became progressively smaller as the number of TBS bouts increased. Thus, while age-dependent effects on LTP expression may be detectable, they are likely to most robustly occur under very specific experimental conditions, with substantial sample sizes. This may limit the routine use of LTP as a measure of synaptic dysfunction in aged mice. Rather, progress in our basic understanding of the aging process in the brain and its potential modulation by drugs may be accelerated by the use of more basic electrophysiological readouts in hippocampal slices (e.g., I–O relationships, PPF).

Together, our studies indicate that the effects of aging on cognition, brain pathology, and synaptic physiology are detectable in C57BL/6 mice despite the considerable challenges that are linked to concurrent age-related changes in hearing, locomotion, and general health. By careful measurement of these noncognitive changes, behavioral protocols can be modified to reduce the impact of these effects. Age-dependent effects on brain function that are unlikely to be due to noncognitive changes were observed in behavioral learning tests (AA), indicators of neuronal function (c-Fos induction in the DG, hippocampal slice physiology), and measures of gliosis (in particular, CD68 staining in the HPF). Hence, this combination of endpoints measuring the effects of aging on cognition, brain pathology, and synaptic physiology are effective in detecting pathology-related decline in C57BL/6 mice.

Models of normal aging are useful as a complementary approach to genetic and other models of human brain pathology and cognitive decline by avoiding some of the challenges linked to these alternative models. For example, genetic models have been challenged, since they typically involve considerable overexpression of proteins bearing mutations that are rarely found in human patients (Bryan et al., 2009). Also, the temporal pattern of changes has been found to translate poorly to humans (Webster et al., 2014). These aspects can limit the translational relevance to clinical populations. Thus, the particular value of studies on normal aging may ultimately be to contribute toward some improvement in bridging the translational gap from preclinical models to cognitive decline in larger human clinical populations.

## Synthesis

The decision was a result of the Reviewing Editor Jeffrey Blaustein and the peer reviewers coming together and discussing their recommendations until a consensus was reached. A fact-based synthesis statement explaining their decision and outlining what is needed to prepare a revision is listed below. The following reviewers agreed to reveal their identity: John Morrison, Jonathan Epp

The reviewers have made some important points, and attention to them would improve the manuscript. In further discussion specifically re: the need for different visual tests, it was agreed that a proper visual test should be concluded in this paper. Although you have made conclusions that vision deficits are unlikely to contribute to the deficits seen with aging, the reviewer points out that there are existing data in the literature to suggest that vision in mice decreases with age. This suggests that it is unlikely that the vision of a 23 month old mouse would be the same as a younger mouse.

Reviewer 1:

This paper represents a comprehensive analysis of potential age-related decrements in mice as measured through numerous cognitive tasks, tests of sensory-motor function, electrophysiological measures of synaptic function, c-fos activation, and immunohistochemical (IHC) analyses of markers for microglia and astrocytes as a reflection of the inflammatory environment. The major strength of the paper is the fact that multiple behavioral tasks are employed along with ephys and microscopy, and the combination of positive and negative results leads to an excellent sense of which measures might be the most useful as screening tools for age-related changes. In addition, for the most part, the authors are careful to note methodological limitations and alternate interpretations, which is particularly useful when considering the potential confounds of age-related sensory and motor defects.

Specific points:

1)While they have multiple data sets from the same animals, they don't present regression analyses linking the data sets. For example, do the ephys results or IHC results correlate on an individual basis with performance on any of the cognitive tasks? Were these done and found to not be informative or were they not pursued? Such analyses have proven to be useful in other studies with multiple data sets.

2)While the behavioral studies seem to be quite rigorous, the morphologic studies are limited by the design, which is very low resolution and not based on measurements individual cells. They acknowledge this limitation, but it would have been nice to see a more rigorous approach.

3)In the Results section (Pages 25-26) they finesse the LTP effect as a "trend towards impaired LTP in aged mice consistent with the impaired cognition..." A p value of 0.11 is just not significant and should not be used to justify this statement. In the Discussion (P. 33) they have a more balanced treatment of the LTP results, but this sentence and any implication that this is a demonstrated deficit should be removed from the Results and the discussion.

Minor points:

1)The designation of DG, CTX, and HPC as ROIs in the Methods (P. 12) and the use of these terms throughout the Results is confusing. First of all, DG is part of HPC, so it is not clear whether HPC always includes DG as well or excludes it. Also, when they say cortex, they mean neocortex (HPC and DG are also cortex) and they should clarify tis distinction.

2)It appears that their CTX measurements are all of neocortex. Is that true? Why did they do this instead of identifying distinct regions with cytoarchitectonic criteria?

3)The links to human aging and transgenic mouse models are overstated in some cases and should be toned down in the discussion.

Reviewer 2:

The central goal of the manuscript under consideration is to assess age related cognitive deficits in mice while controlling for potential confounding deficits such as sensory and motor impairments. The authors identify age related impairments in both spatial memory and active avoidance learning. Acoustic startle responses suggest an impairment in hearing in aged animals and the authors argue that a lack of deficit in a visual placing task indicates normal vision in the aged mice. The authors go on to show some physiological changes that may be responsible for the cognitive deficits including increased gliosis, decreased c-fos activity and decreased PPF in the hippocampus.

In general, I feel quite positive about the work presented here and the approach taken to separate cognitive and sensory deficits in aging. My main concern with the manuscript is that the authors have fallen critically short in their assessment of visual capabilities. Where as the test of auditory function allows for a refined assessment of how well the mice can hear (by testing at increasing dB), the test of visual function is little more than a binary measure of whether the mice are blind or not. The use of this test is somewhat conceptually similar to testing the mice only with the lowest acoustic intensity and surmising that there were no auditory deficits. The addition of a more sensitive visual assessment is crucial here because the authors tailor their active avoidance protocol to use a visual only approach but have not in my opinion ruled out deficits in visual ability in aged mice. A visual acuity test similar to that used in the following paper would be a preferable strategy.

Visual detection, pattern discrimination and visual acuity in 14 strains of mice

A. A. Wong and R. E. Brown*

Genes, Brain and Behavior (2006) 5: 389-403
